# Increased proliferation and neuronal fate in prairie vole brain progenitor cells cultured in vitro: effects by social exposure and sexual dimorphism

**DOI:** 10.1186/s13293-023-00563-2

**Published:** 2023-11-02

**Authors:** Daniela Ávila-González, Italo Romero-Morales, Lizette Caro, Alejandro Martínez-Juárez, Larry J. Young, Francisco Camacho-Barrios, Omar Martínez-Alarcón, Analía E. Castro, Raúl G. Paredes, Néstor F. Díaz, Wendy Portillo

**Affiliations:** 1 Departamento de Neurobiología Conductual y Cognitiva, Instituto de Neurobiología, Universidad Nacional Autónoma de México, Juriquilla, Querétaro Mexico; 2 Departamento de Fisiología y Desarrollo Celular, Instituto Nacional de Perinatología Isidro Espinosa de los Reyes, Mexico City, Mexico; 3 Silvio O. Conte Center for Oxytocin and Social Cognition, Center for Translational Social Neuroscience, Emory National Primate Research Center, Department of Psychiatry and Behavioral Sciences, Emory University, Atlanta, USA; 4 Escuela Nacional de Estudios Superiores Juriquilla, Universidad Nacional Autónoma de México, Querétaro, Mexico

**Keywords:** Pair bond, Neurogenesis, Gliogenesis, Sociosexual behavior, Estradiol, Brain-derived neurotrophic factor, Prolactin, Oxytocin, Progesterone

## Abstract

**Background:**

The prairie vole (*Microtus ochrogaster*) is a socially monogamous rodent that establishes an enduring pair bond after cohabitation, with (6 h) or without (24 h) mating. Previously, we reported that social interaction and mating increased cell proliferation and differentiation to neuronal fate in neurogenic niches in male voles. We hypothesized that neurogenesis may be a neural plasticity mechanism involved in mating-induced pair bond formation. Here, we evaluated the differentiation potential of neural progenitor cells (NPCs) isolated from the subventricular zone (SVZ) of both female and male adult voles as a function of sociosexual experience. Animals were assigned to one of the following groups: (1) control (Co), sexually naive female and male voles that had no contact with another vole of the opposite sex; (2) social exposure (SE), males and females exposed to olfactory, auditory, and visual stimuli from a vole of the opposite sex, but without physical contact; and (3) social cohabitation with mating (SCM), male and female voles copulating to induce pair bonding formation. Subsequently, the NPCs were isolated from the SVZ, maintained, and supplemented with growth factors to form neurospheres in vitro.

**Results:**

Notably, we detected in SE and SCM voles, a higher proliferation of neurosphere-derived Nestin + cells, as well as an increase in mature neurons (MAP2 +) and a decrease in glial (GFAP +) differentiated cells with some sex differences. These data suggest that when voles are exposed to sociosexual experiences that induce pair bonding, undifferentiated cells of the SVZ acquire a commitment to a neuronal lineage, and the determined potential of the neurosphere is conserved despite adaptations under in vitro conditions. Finally, we repeated the culture to obtain neurospheres under treatments with different hormones and factors (brain-derived neurotrophic factor, estradiol, prolactin, oxytocin, and progesterone); the ability of SVZ-isolated cells to generate neurospheres and differentiate in vitro into neurons or glial lineages in response to hormones or factors is also dependent on sex and sociosexual context.

**Conclusion:**

Social interactions that promote pair bonding in voles change the properties of cells isolated from the SVZ. Thus, SE or SCM induces a bias in the differentiation potential in both sexes, while SE is sufficient to promote proliferation in SVZ-isolated cells from male brains. In females, proliferation increases when mating is performed. The next question is whether the rise in proliferation and neurogenesis of cells from the SVZ are plastic processes essential for establishing, enhancing, maintaining, or accelerating pair bond formation.

**Highlights**

1. Sociosexual experiences that promote pair bonding (social exposure and social cohabitation with mating) induce changes in the properties of neural stem/progenitor cells isolated from the SVZ in adult prairie voles.
2. Social interactions lead to increased proliferation and induce a bias in the differentiation potential of SVZ-isolated cells in both male and female voles.
3. The differentiation potential of SVZ-isolated cells is conserved under in vitro conditions, suggesting a commitment to a neuronal lineage under a sociosexual context.
4. Hormonal and growth factors treatments (brain-derived neurotrophic factor, estradiol, prolactin, oxytocin, and progesterone) affect the generation and differentiation of neurospheres, with dependencies on sex and sociosexual context.
5. Proliferation and neurogenesis in the SVZ may play a crucial role in establishing, enhancing, maintaining, or accelerating pair bond formation.

**Supplementary Information:**

The online version contains supplementary material available at 10.1186/s13293-023-00563-2.

## Plain language summary

In this study, researchers evaluated whether social interactions and copulation induce changes in the proliferation and differentiation of neural progenitor cells in adult male and female voles using an in vitro neurosphere formation assay. The following groups were assigned: control animals without any exposure to another vole outside their litter, another group with social exposure consisting of sensory exposure to a vole of the opposite sex and a third group with social cohabitation and copulation. Forty eight hours after social interactions, cells were isolated from the neurogenic niche subventricular zone (SVZ) and cultured to assess their self-renewal and proliferation abilities to form neurospheres. The results showed in the social interaction groups, a greater number and growth of neurospheres in both males and females. Differentiation capacity was assessed by immunodetection of MAP2 and GFAP to identify neurons or glia, respectively, arise from neurospheres, with an increase in neuronal fate in groups with social interaction. In the second part of the study, the researchers analyzed the effect of different hormone and growth factor treatments and found that the response in both proliferation and differentiation potential may vary depending on the sociosexual context or sex. This study suggests that social interactions leading to pair bond formation alter the properties of SVZ cells, whereby proliferation and neurogenesis may have an impact on the establishment and maintenance of pair bonding.

**Supplementary Information:**

The online version contains supplementary material available at 10.1186/s13293-023-00563-2.

## Background

Pair bonding is an enduring preferential association formed between two sexually mature adults, with implications for subjects' mental and physiological health [1–3]. In humans, the pair bonding dynamic is extraordinarily complex because of social and cultural factors, complicating its investigation at the neurobiological level. The prairie vole (*Microtus ochrogaster)* is one of the few socially monogamous mammals that has been used extensively to elucidate the neurobiological basis of pair bonding [4]. Pair-bonded voles exhibit preferential contact and mating with their partner over a stranger, referred to as partner preference, which is used in the laboratory to assess pair bonding. Cohabitation with mating for 6 h or social exposure for 24 h induces partner preference mediated through vasopressin, oxytocin, and dopamine neurotransmission in a sexually dimorphic manner [5–7].

Adult neurogenesis is a crucial process of neural plasticity that leads to behavioral changes and plays a fundamental role in sexual behavior, pregnancy and maternal behavior [8–11]. The subventricular zone (SVZ) is the main neurogenic niche of the adult mammalian brain, where neural stem cells (type B cells) divide and give rise to intermediate progenitors (type C cells) that produce migrating neuroblasts (type A cells). Neuroblasts move along the rostral migratory stream (RMS) towards the olfactory bulb (OB), where they differentiate into interneurons [12–17]. Interestingly, SVZ neural stem cells can be cultured, recapitulated in vitro for in vivo neurogenesis and generate new cells in the OB when transplanted into the SVZ in vivo [15].

Adult neurogenesis depends on internal and external environmental cues. Indeed, sexual and social behaviors regulate the proliferation, maturation and survival of new neurons in the OB and dentate gyrus (DG) of the hippocampus [18]. Exposure to sexually relevant cues and sexual behavior in male and female rats and mice increases the number of new cells in the OB and DG [19–23].

Thus, adult neurogenesis plays a fundamental role in sexual behavior, social and sexual partner recognition and mate choice. Indeed, male mice in which adult neurogenesis was eliminated showed a decrease in sexual behavior [24]. In male rats, adult neurogenesis inhibition reduces intromission frequency and males do not ejaculate [25]; whereas female mice treated with cytosine arabinose (Ara-c, antimitotic) had decreased OB and DG neurogenesis. Ara-c treatment impairs the ability to recognize the male with whom they mate [26]. In male hamsters, newborn cells in the OB are activated in response to vaginal secretion. In contrast, in female mice, dominant male pheromones increased cell proliferation in the SVZ and exposure to male pheromones augmented the number of new neurons that survived in the OB. Interestingly, newborn cells are necessary to prefer socially dominant males [21]. Thus, new neurons may be involved in sexual behavior, partner recognition, and mate selection [18].

In prairie voles, OB plays a relevant role in socio-sexual behavior. Males lesioned in this neuronal region show decreased social behavior, do not mate, and cannot show partner preference, whereas female voles lesioned display reduced contact with males and do not prefer their sexual partners [27, 28]. Therefore, these studies highlight the relevance of OB in sexual behavior and mate preferences.

The influence of sexual experience on neurogenesis in voles has also been investigated. In females, estrus induction by exposure to unrelated familiar males increases cellular proliferation (90%) in the RMS and the cells committed to the neuronal lineage (80%) [29]. In addition, cohabitation with a male increased cell proliferation in the amygdala and hypothalamus, but had no effect on the SVZ and DG [30]. Our research group demonstrated an increase in cell proliferation and differentiation into new immature neurons in males that were exposed to a female without physical contact and in voles that socially cohabited with mating [31]. Cell survival studies have demonstrated that in female voles, social cohabitation with mating increases the number of new cells and mature neurons in the glomerular layer of the main OB and the number of new cells in the suprapyramidal layer of the DG. However, social cohabitation with mating decreases the number of new cells and neurons in the anterior glomerular layer of accessory OB. In male voles, social cohabitation with mating and social exposure to females decrease the number of new cells and neurons in the glomerular layer of the main OB [32]. Thus, social cohabitation with mating and social exposure to an opposite-sex vole modulate cell survival in the OB and DG. These new cells may be involved in mate recognition, which is a fundamental process in pair bonding.

Previously, our research group demonstrated that cells isolated from neurogenic niches in prairie voles formed aggregates called neurospheres, which can be maintained under controlled culture conditions [33]. The assay of neurospheres has been demonstrated to be a valuable in vitro technique for investigating the properties of neural stem cells (NSCs) and neural progenitors cells (NPCs), such as proliferation and differentiation potential, as well as the extrinsic and intrinsic factors that modulate these processes [34, 35].

Here, we studied how social interactions such as cohabitation and mating affect the differentiation and proliferation of cells isolated from the SVZ in male and female voles.

The proliferative potential of neuronal progenitor cells from the SVZ was evaluated based on the number and size of the neurospheres. In contrast, migration was determined by the average distance between the radius of the inner and outer circumferences of the neurosphere outgrowth. Nestin was used to identify neural stem cells, and the 5-ethinyl-2-deoxyuridine (EdU) assay was used to determine cell proliferation. Finally, we evaluated the differentiation potential of doublecortin (DCX, neuroblast), microtubule-associated protein 2 (MAP2, mature neurons), and glial fibrillary acid protein (GFAP, glial cells).

Additionally, the effects of brain-derived neurotrophic factor (BDNF) and hormones [estradiol (E2), progesterone (P4), prolactin (PRL), and oxytocin (OXY)] on neurosphere proliferation and differentiation were evaluated.

## Materials and methods

### Experimental groups

This study was approved by the Research Ethics Committees of the Instituto de Neurobiología (072), Universidad Nacional Autónoma de México and Instituto Nacional de Perinatología (2018-1-163). Adult female and male prairie voles (3–4 months old) were obtained from a colony whose founding members were donated by Dr. Larry J. Young at the Emory University. Voles were maintained in a 14 h light-10 h dark cycle at, 20–25 °C conditions and fed ad libitum with sunflower seeds, oats, and a Laboratory Rabbit Diet High Fiber (LabDiet, 5326). Prairie voles were housed in 40 × 20 × 20 cm acrylic cages with pine chip bedding and brown paper as the nesting material. Female voles are induced ovulators and do not display a spontaneous cycle; however, exposure to non-familiar males leads to reproductive activation, also called behavioral estrous [36]. The experimental subjects were gonadally intact without hormone replacement and randomly assigned to three groups: (1) Control (Co) group; sexually virgin subjects were placed alone in clean individual acrylic boxes (40 × 20 × 20 cm) for 48 h. (2) Social exposure (SE) group, in which male and female voles were placed in acrylic cages (40 × 20 × 20 cm) divided into two equal compartments by a plastic screen with small holes. Under this condition, they were exposed for 48 h to visual, olfactory, and auditory stimuli from a vole of the opposite sex without physical contact. (3) Social cohabitation with mating (SCM) group: male and female mice were housed together in acrylic cages (40 × 20 × 20 cm) and were able to cohabitate and copulate for 48 h to induce pair bonding. During the experiments, all animals were provided with food and water ad libitum*.* At the end of the experiments, the subjects were euthanized with an overdose of pentobarbital (6.3 mg/vole) by intraperitoneal (IP) administration, and the whole brains were obtained to dissect the SVZ tissue.

### Isolation and culture of SVZ-derived cells

SVZ tissue dissection was performed as previously described [33]. Briefly, the whole brain was collected in Petri dishes, placed on an ice-cold surface, and the SVZ was dissected using a stereoscope. The tissue was washed with a solution of 2 mM HEPES (Sigma-Aldrich, H3375), 20 mM glucose (Sigma-Aldrich, G7021), and 25 mM NaHCO3 (Sigma-Aldrich, S5761). Subsequently, the tissue was incubated in an enzymatic solution composed of DMEM-F12 with dispase (0.875 mg/mL) (Thermo Fisher Scientific/Gibco, 17105041) and collagenase type IV (0.9 mg/mL) (Thermo Fisher Scientific/Gibco, 17104019) at 37 °C for 20 min. At the end of the incubation period, the tissue was triturated by pipetting using 1000 μL tips. The cell suspensions were centrifuged at 200 × g for 4 min at room temperature and washed with N2 medium [DMEM-F12 (Thermo Fisher Scientific/Gibco, 11330032) with N2 supplement (Thermo Fisher Scientific/Gibco, 17502048), l-glutamine (Thermo Fisher Scientific/Gibco, 35050061), and antibiotic–antimycotic (Thermo Fisher Scientific, Gibco, 15240062)] to remove dispase and collagenase. The isolated cells were resuspended in B27 medium [Neurobasal medium (Thermo Fisher Scientific/Gibco, 21103049), B27 supplement (Thermo Fisher Scientific/Gibco, 17504044), L-glutamine, and antibiotic–antimycotic] supplemented with epidermal growth factor (EGF, 10 ng/mL) (PeproTech, AF-100-15), and fibroblast growth factor 2 (FGF2, 10 ng/mL) (PeproTech, AF-100-18B) for 48 h without medium change on low-adherence plates. The whole culture medium with the growth factors was changed every third day, and on the second day, the growth factors were added at the same concentration until the formation of neurospheres after eight days (D8). At D15, the neurospheres were seeded in plates treated with poly-L-ornithine (Sigma-Aldrich, P3655) and laminin (Merck Millipore, CC095) to allow the adherent expansion of progenitor cells, with a B27 medium supplemented with growth factors. The cells were maintained for ten days in B27 medium without the addition of growth factors for the differentiation assays.

### Immunostaining

Neurospheres and adherent cells were fixed with 4% PFA, washed three times with 1 × PBS and then permeabilized with 0.3% Triton (Sigma-Aldrich, T8787) for 20 min, followed by an incubation period in blocking solution [5% bovine serum albumin (BSA) (Biowest, P6156) and 0.05% Tween (Sigma-Aldrich, P1379)] for 30 min. The samples were incubated overnight at 4 °C with the following primary antibodies in blocking solution: mouse anti-nestin, 1:200 (Genetex, GTX30671), guinea pig anti-double cortin (DCX), 1:4000 (Merck Millipore, AB2253), rabbit anti-microtubule associated protein 2 (MAP2) (Genetex, GTX50810), and mouse anti-glial fibrillary acid protein (GFAP), 1:500 (Sigma-Aldrich, G3893). The next day, the samples were washed three times with 1 × PBS (Thermo Fisher Scientific/Gibco, 10010023). Secondary antibodies coupled to fluorophores [Alexa 488 (Thermo Fisher Scientific, A-11029 and A-11073) and Alexa 568 (Thermo Fisher Scientific, A-11036]) in blocking solution at a concentration of 1:1000 were added and incubated for one hour at room temperature. The samples were washed three times with 1 × PBS and 4′, 6-diamino-2-phenylindole (5 µg/mL in 1 × PBS) (Thermo Fisher Scientific, D1306) was added to stain the nuclei. Primary antibodies have been validated in previous studies, and secondary antibody non-specificity was controlled by omitting the primary antibody (data not shown).

Immunofluorescence was visualized under an epifluorescence microscope (Olympus IX -81), and microphotographs were obtained using a CCD camera (Hamamatsu, ORCA-Flash). For each immunostaining marker, the integrated density (IntDen) level was determined using ImageJ software. The corrected total cell fluorescence (CTCF) intensity was calculated using the following formula: CTCF = IntDen − (area of selected cell × mean fluorescence of background readings). The data were normalized to the control groups and plotted as Relative Fluorescence [37].

### Cell proliferation assays

The adhered neurosphere-derived cells were incubated in B27 medium supplemented with the thymidine analog 5-ethinyl-2´-deoxyuridine (EdU, 10 µM) (Thermo Fisher Scientific/Invitrogen, C10337) for one hour at 37 °C. EdU is a nucleoside analog of thymidine that is incorporated into DNA during active DNA synthesis. Therefore, it can be used as a marker for proliferating cells. At the end of the incubation, the EdU labeling treatment was removed, and the cells were fixed with 4% PFA for 15 min, followed by two washes with 1 × PBS-3% BSA. The samples were permeabilizated with Triton 0.5% in 1 × PBS for 20 min at room temperature, followed by two washes with 1 × PBS-3% BSA. To detect EdU + cells, they were incubated with a reaction cocktail (Click-iT EdU reaction buffer, CuSO4, Alexa Fluor-488, and Click-iT EdU additive buffer [all reagents from Thermo Fisher Scientific, C10337]) for 30 min at room temperature and protected from light. The percentage of EdU + cells was calculated by dividing the number of EdU + /Nestin + cells by the total number of DAPI + cells/field and multiplying the result by 100. We normalized the data with DAPI staining for ROI analyses. Therefore, in the following analysis, instead of ROI determination, we counted the percentage of positive cells based on the total number of DAPI + cells per field. In the count of proliferating cells, we showed the data with the Nestin +/EdU + double label to confirm that they were NPCs and not misinterpreted as non-neural cells.

### Statistical analysis

The data were analyzed using GraphPad Prism, SigmaPlot 11.0, and R programming language (R Core Team, 2023), displaying the graphs with the mean ± standard error. All data were analyzed using the Shapiro–Wilk normality test and exhibited a normal distribution. For statistical analyses, six to seven neurospheres from each vole were evaluated, and the mean was used to minimize variability.

**Fig. 1:**
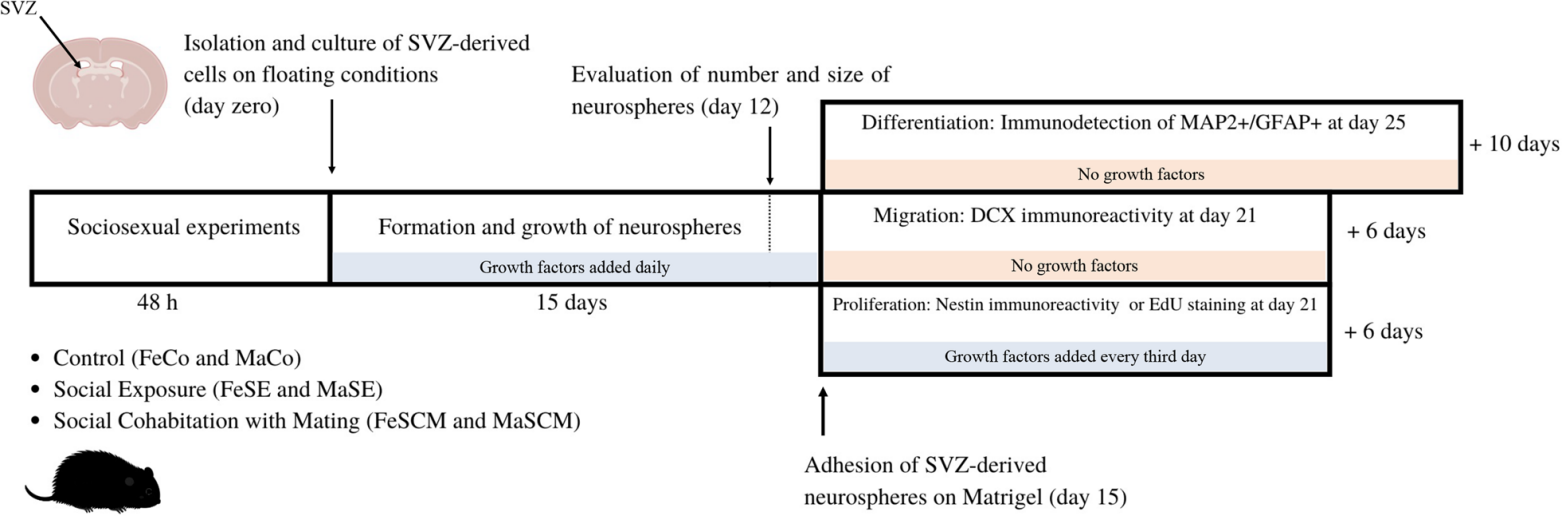
Experimental strategy to evaluate though the neurosphere assay both proliferation and differentiation potentials of SVZ-isolated cells of control, social exposure, and social cohabitation with mating voles

**Fig. 2:**
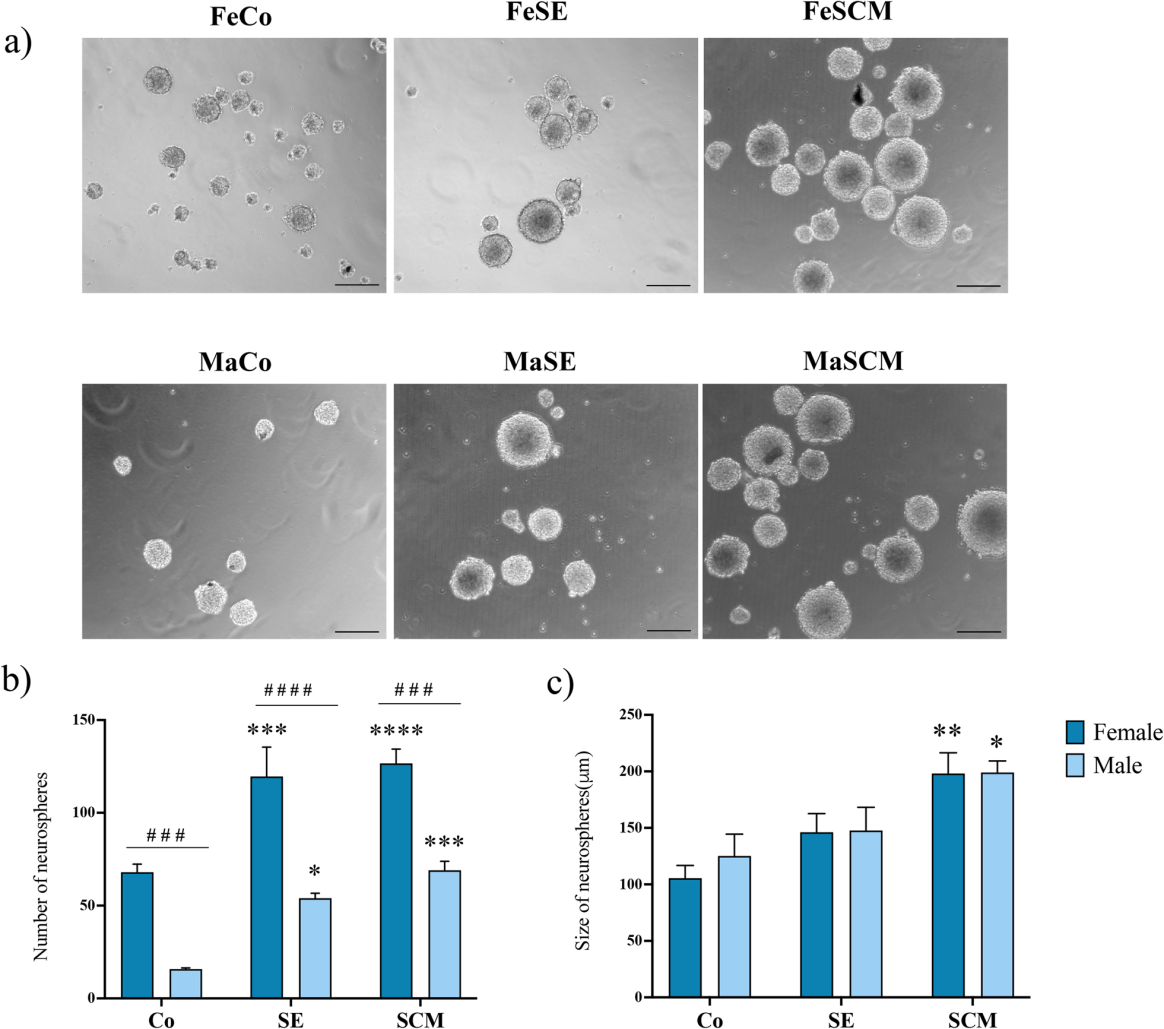
Social exposure and social cohabitation with mating increase the number and size of SVZ-derived neurospheres from adult prairie voles. **a** Representative micrographs of neurospheres derived from SVZ-isolated cells of the adult female (Fe) and male (Ma) prairie vole in control (Co), social exposure (SE) and social cohabitation with mating (SCM) groups. Scale bar, 200 µm. **b** Number of neurospheres obtained from SVZ in both Fe and Ma voles. Data were analyzed with a two-way ANOVA followed by a Tukey’s post hoc test. n = 7 subjects per group of each sex. **c** Size (diameter) of neurospheres measured 12 days after initiation of culture. Data were analyzed with a two-way ANOVA followed by Tukey’s post hoc test. n = 6 per group of each sex, seven neurospheres per individual were analyzed. Different from Co group *p < 0.05, **p < 0.01, ***p < 0.001, ****p < 0.0001. Sex differences in the same sociosexual group ^###^*p* < 0.001, ^####^*p* < 0.0001

**Fig. 3:**
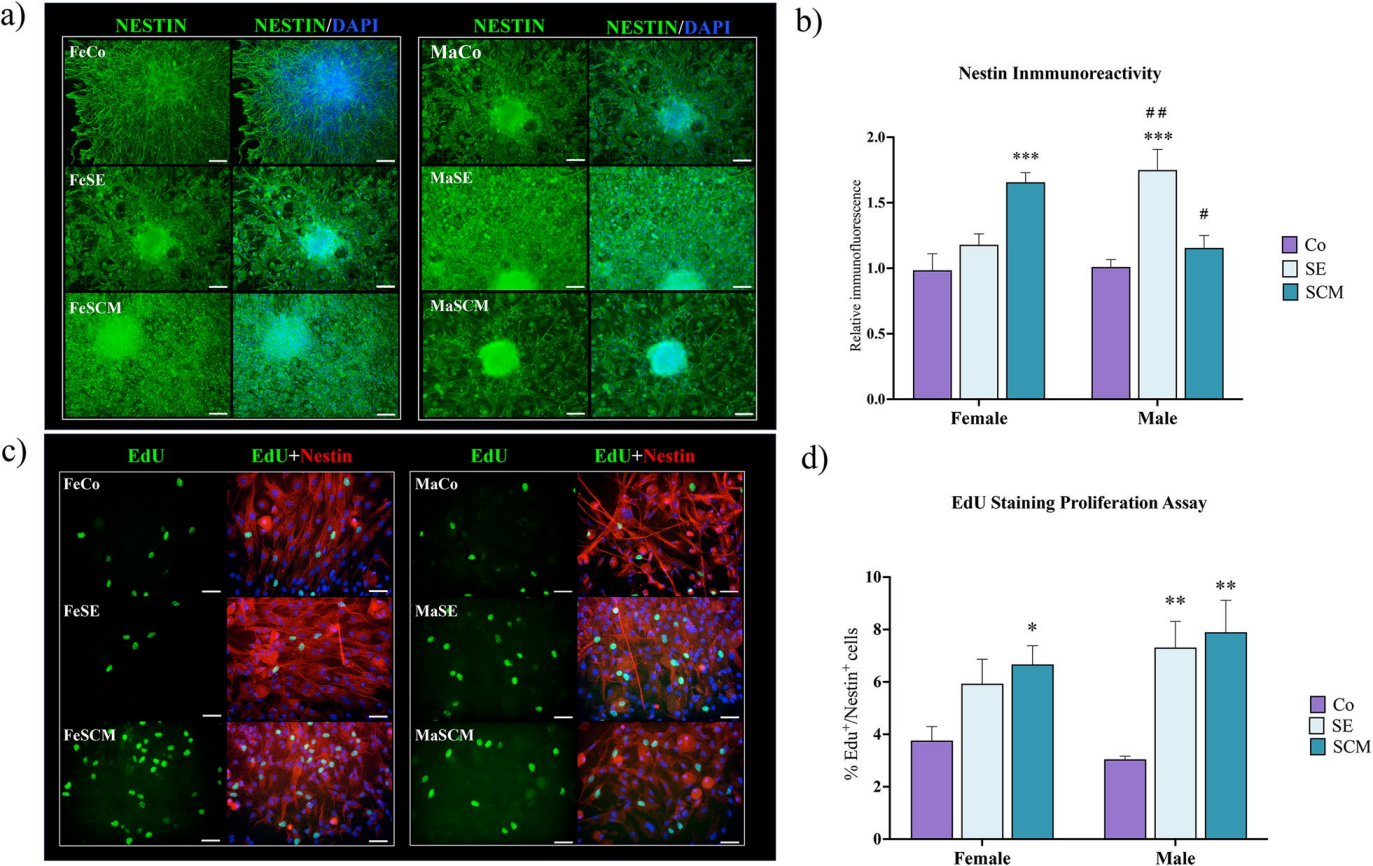
Increased proliferation in the cellular outgrowth of attached neurospheres derived from SVZ-isolated cells in the adult vole previous sociosexual stimuli. **a** Representative fluorescence microscopy images of Nestin + (green) cells from SVZ-derived neurospheres of control (Co), social exposure (SE) and social cohabitation with mating (SCM) groups in both female (Fe) and male (Ma) adult voles. Nuclei were stained with DAPI (blue). Individual nuclei are not visible because neurospheres can comprise several hundred cells. Scale bars = 50 µm. **b** Determination of immunoreactivity to Nestin in undifferentiated cells from SVZ-derived neurospheres. Relative fluorescence was analyzed with a one-way ANOVA followed by Tukey’s post hoc test. FeCo and FeSE (n = 8 per group) and FeSCM (n = 9); for males n = 9 per group. Six neurospheres per individual were analyzed. **c** Representative fluorescence microscopy images of EdU + (green) and Nestin + (red) cells from SVZ-derived neurospheres of Co, SE and SCM groups in both female (Fe) and male (Ma) adult voles. Nuclei were stained with DAPI (blue). Scale bars = 50 µm. **d** Percentage of EdU + cells in undifferentiated cells from SVZ-derived neurospheres. The data was analyzed with a one-way ANOVA followed by Tukey’s post hoc* test*. Females n = 10 per group; MaCo and MaSCM (n = 10 per group) and for MaSE (n = 9). Six neurospheres per individual were analyzed. Different from the Co group *p < 0.05, **p < 0.01 and ***p < 0.001. Sex differences in the same sociosexual group ^#^*p* < 0.05, ^##^*p* < 0.01

**Fig. 4:**
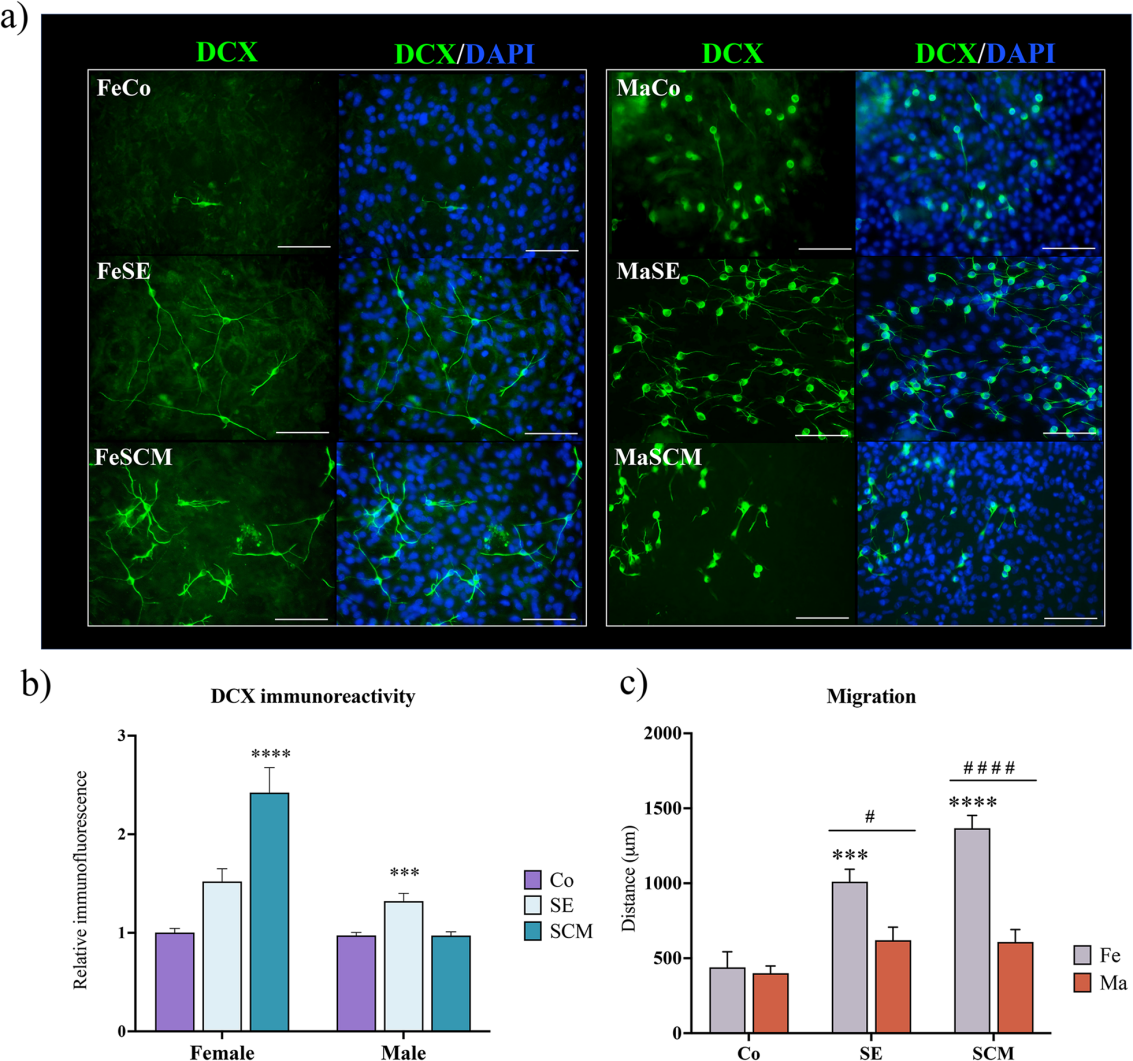
Social exposure affects DCX and migration in cells derived from neurospheres grown up from SVZ-isolated adult voles. **a** Representative fluorescence microscopy images of DCX + (green) cells in the outgrowth of attached SVZ-derived neurospheres of control (Co), social exposure (SE) and social cohabitation with mating (SCM) groups in both female (Fe) and male (Ma) adult voles. Nuclei were stained with DAPI (blue). Scale bars = 100 µm. **b** Relative immunofluorescence of DCX + cells in SVZ-derived neurospheres. The data for each sex group was analyzed with a one-way ANOVA followed by Tukey’s post hoc* test*. FeCo, FeSCM (n = 8 per group), FeSE (n = 7); MaCo and MaSCM (n = 8 per group) and MaSE (n = 7). Six neurospheres per individual were analyzed. **c** Migration (measured distance from the edge of the neurospheres to the cell outgrowth) of cells from SVZ-derived neurospheres. To measure relative migration, light phase-contrast photomicrographs were taken and the average distance between the radius of the inner and outer circumference of the neurosphere outgrowth was calculated. Data was analyzed with a two-way ANOVA followed by Tukey´s post hoc* test*. n = 6 per group in each sex, six neurospheres per individual were analyzed. Different from Co group ***p < 0.001, ****p < 0.0001. Sex differences in the same group ^**#**^*p* < 0.05, ^**####**^*p* < 0.0001

**Fig. 5:**
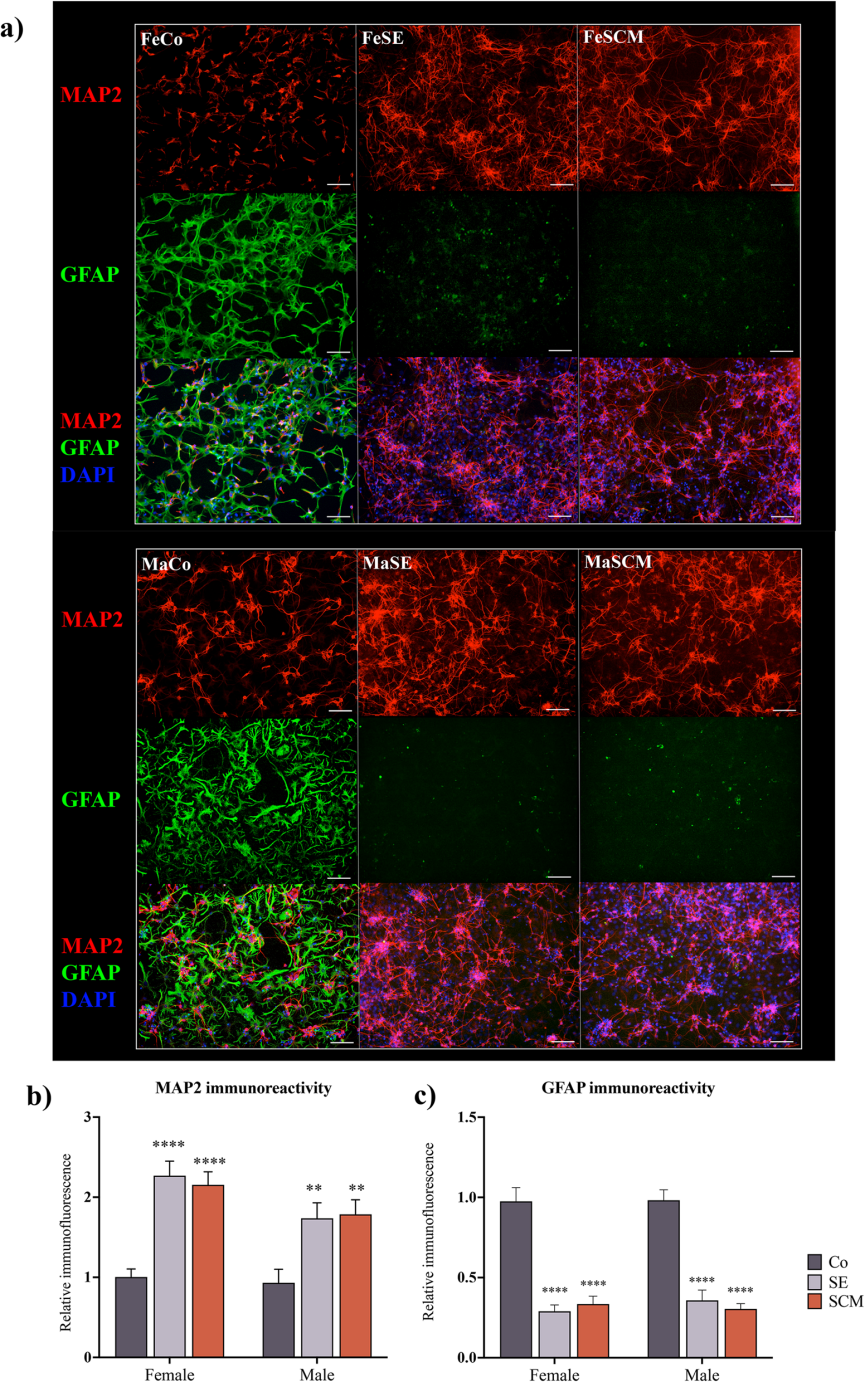
Cells isolated from SVZ have a greater potential for differentiation towards neuronal lineage and a decrease towards glial lineage in sociosexual voles. **a** and **b** Representative fluorescence microscopy images of mature neurons MAP2 + (red) and glial GFAP + (green) cells from SVZ-derived neurospheres of control (Co), social exposure (SE) and social cohabitation with mating (SCM) groups in female (Fe) and male (Ma) adult voles. Nuclei were stained with DAPI (blue). Scale bars = 50 µm. **b** Determination of immunoreactivity to MAP2 in differentiated cells from SVZ-derived neurospheres of Co, SE and SCM groups in both Fe and Ma adult voles. Females n = 10 per group, MaCo and MaSE (n = 10) and MaSCM (n = 9). **c** Quantification of immunoreactivity to GFAP in differentiated cells from SVZ-derived neurospheres of Co, SE and SCM groups. FeCo and FeSCM (n = 10 per group), FeSE (n = 9); MaCo and MaSE (n = 10) and MaSCM (n = 9). The MAP2 and GFAP data for each sex group (Fe or Ma) was analyzed with a one-way ANOVA followed by Tukey’s post hoc test. Six neurospheres per individual were analyzed. Different from Co group **p < 0.005, ****p < 0.0001

In the first part (Fig. 1), statistical differences in the number and size of neurospheres (Fig. 2), Edu + /Nestin + percentage data (Fig. 3d), and migration (Fig. 4c) were determined by two-way ANOVA (sex and sociosexual group as independent variables) followed by Tukey’s post hoc test (*p* ≤ 0.05). Nestin (Fig. 3b), DCX (Fig. 4b), MAP2 (Fig. 5b), and GFAP (Fig. 5c) relative immunofluorescence data were normalized to the same sex control group (FeCo and MaCo); therefore, comparisons were performed only between sociosexual groups. Therefore, these data were analyzed using one-way ANOVA (social group as an independent variable), followed by Tukey’s post hoc test (*p* ≤ 0.05).

**Fig. 6:**
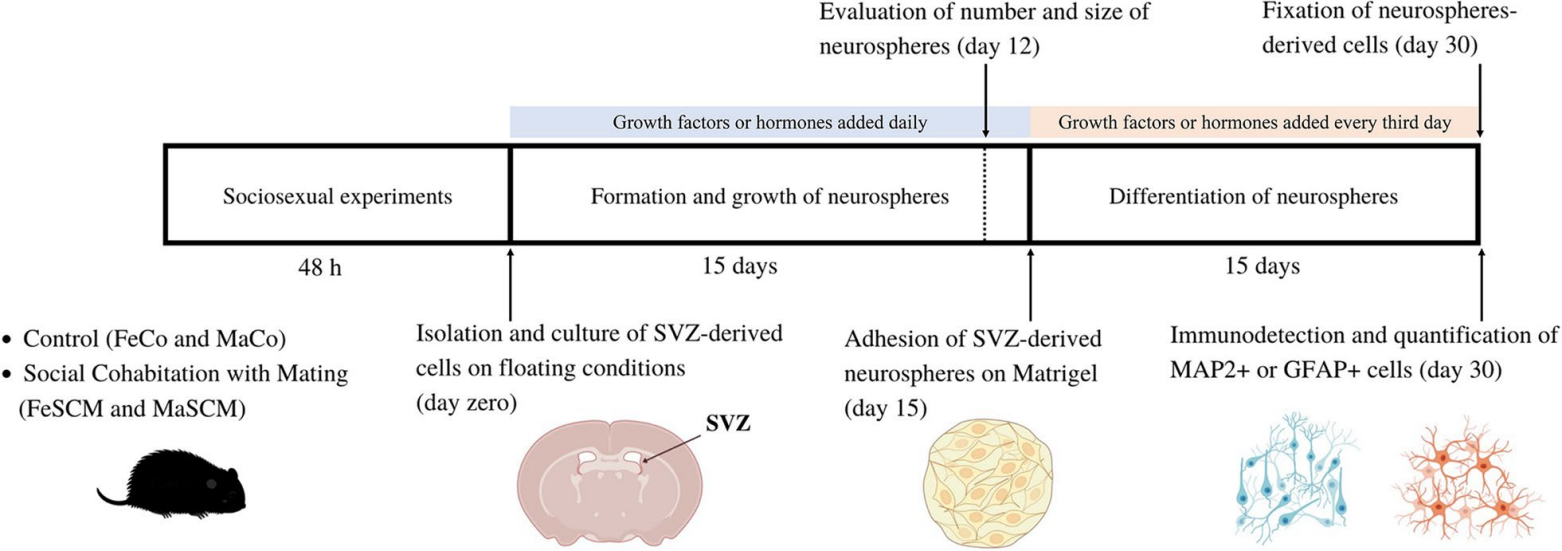
Experimental strategy to analyze the effect of different treatments of growth factors or hormones on the formation and differentiation of neurospheres derived from the SVZ of control (Co) and social cohabitation with mating (SCM) voles

In the second part (Fig. 6), BDNF, E2, PRL, OXY, and P4 data on the number and growth of neurospheres and percentage of MAP2 + or GFAP + cells were analyzed with the Shapiro–Wilk normality test and exhibited a normal distribution. Statistical differences for these data were determined by two-way ANOVA followed (sex and sociosexual group as independent variables) by Tukey’s post hoc test (*p* ≤ 0.05).

### Alternative treatments to generate SVZ-derived neurospheres

In addition to conventional treatment (EGF 20 ng/mL plus FGF2 20 ng/mL) to form SVZ-derived neurospheres, isolated cells were cultured under low-adherence-conditions with the following treatments: EGF (20 ng/mL), FGF2 (20 ng/mL), BDNF human (Tocris, 2837) (20 ng/mL, 50 ng/mL and 20 ng/mL plus EGF 20 ng/mL); (Thr4, Gly7)-Oxytocin (Bachem, 4,013,837.001) (0.5 µM, 1 µM and 0.5 µM plus EGF 20 ng/mL); progesterone (Sigma Aldrich, P7556) (1 µM, 2 µM and 1 µM plus EGF 20 ng/mL); prolactin (Peprotech, 315–16) (50 ng/mL, 100 ng/mL, 200 ng/mL and 100 ng/mL plus EGF 20 ng/mL), estradiol benzoate (Sigma Aldrich) (0.5 µM, 1 µM, 2 µM and 1 µM plus EGF 20 ng/mL) and without any growth factor or hormone. Cells isolated from four animals of each sex were used for the BDNF, OXY, FGF2, and EGF groups, and another four animals of each sex were used for the P4, PRL, and E2 groups.

The culture medium was changed every third day, and growth factors or hormones were added daily. On day 12, the total number and diameter of the neurospheres were quantified for each treatment. On day 15, cellular aggregates were cultured on plates treated with poly-L-ornithine and laminin to adhere to the cells. For differentiation, the adhered cells were maintained for 15 days in B27 medium supplemented with each of the aforementioned treatments. The medium supplemented with growth factors or hormones was changed every three days. Thirty days after the initial isolation, cells were fixed with 4% PFA, and the previously mentioned immunostaining protocol was carried out to detect MAP2 or GFAP. For these experiments, quantification by relative fluorescence was ruled out because cell density was not homogeneous between the different treatment groups, so we determined the percentage of MAP2 + or GFAP + cells.

As previously reported, cell nuclei were labeled with the nuclear marker 4’,6-diaminofenyl-indol DAPI and their counting was performed using Fiji-Image J software, according to previously established morphometric parameters and reported [31]. Briefly, 8-bits images from different experimental conditions were processed; the nuclear size (50-infinity) and circularity (0.05–1.00) were established using the Analyze Particle tool to exclude non-specific backgrounds. The nuclei located in the image corners and borders were not quantified. To obtain the percentage of MAP2 + or GFAP + cells for each treatment, we used the following equation: % of positive cells = [number of positive cells to a specific marker/total number of cells (DAPI stained nuclei)] × 100.

## Results

### Number and size of SVZ-derived neurospheres

We performed a neurosphere formation assay to evaluate the proliferation and differentiation of cells derived from the SVZ of adult voles. Because 6 h of mating or 24 h of cohabitation are sufficient to form a pair bond in both sexes, our behavioral testing lasted 48 (Experimental timeline in Fig. 1). Cell aggregates were observed before D8. The cultures were maintained in a culture medium supplemented with FGF2 for additional four days (D12) and the total number of neurospheres obtained per group was quantified. Representative photomicrographs of SVZ-derived neurospheres from female (Fe) and male (Ma) voles from the control (FeCo, MaCo), Social Exposure (FeSE, MaSE), and Social Cohabitation with Mating (FeSCM, MaSCM) groups are presented in Fig. 2a.

We detected a significant change in the number of SVZ-derived neurospheres due to the effects of sociosexual experience (F _(2, 36)_ = 29.23, *p* < 0.0001) and sex (F _(1, 36)_ = 85.46, *p* < 0.0001), although no differences in sex and sociosexual interaction were found (F _(2, 36)_ = 0.3788, *p* = 0.6874). Post hoc analysis revealed that the SE and SCM groups for each sex presented a greater number of SVZ-derived neurospheres than their respective control group: FeCo vs. FeSE (*p* < 0.0005), FeCo vs. FeSCM (*p* < 0.0001), MaCo vs. MaSE (*p* < 0.0152), and MaCo vs. MaSCM (*p* < 0.003). In addition, when comparing between sexes, the number of neurospheres decreased in males when compared to females in each sociosexual group (FeCo vs. MaCo, *p* < 0.0004; FeSE vs. MaSE, *p* < 0.0001; FeSCM vs. MaSCM, *p* < 0.001) (Fig. 2b). The growth of neurospheres was measured at D12, with significant differences found in sociosexual factor (F_(2, 36)_ = 13.03, *p* < 0.0001), but not in sex. In female groups, we did not find differences in the size of neurospheres in FeCo vs. FeSE, whereas there was an increase in the size in FeSCM than FeCo (*p* < 0.0043) (Fig. 2c). Regarding male groups, in MaSCM there was an increase in size with respect to MaCo (*p* < 0.0323).

### Analysis of the proliferation potential of cells of SVZ-derived neurospheres

Once the neurospheres were attached at D15, they were randomly divided into experimental subgroups; some were used to assess proliferation and others to identify their differentiation potential (experimental timeline in Fig. 1). For proliferation, the cells were maintained in a medium supplemented with FGF2 and EGF for an additional six days (21 days after SVZ isolation), which has a mitogenic effect on the NSCs of rodents and humans [38–40]. The cells that migrated out of neurospheres were identified through Nestin, a marker of an undifferentiated state that precedes the exit of the cell cycle and the commitment to specific mature cell lineages [41, 42] (Fig. 3a).

We found a significant difference in the immunoreactivity to Nestin between sociosexual groups (F_(2,43)_ = 11.92, *p* < 0.0001) and with its interaction with sex factor (F_(2,43)_ = 13.47, *p* < 0.0001). FeSCM showed an increase in Nestin signal compared to FeCo (*p* = 0.0003), whereas SVZ-derived neurospheres of MaSE presented an increase in the relative intensity of Nestin immunoreactivity compared to MaCo (*p* = 0.0001) (Fig. 3b). When comparing between sexes, in the SE groups there was greater reactivity to Nestin in males than females (*p* = 0.0066), while in SCM animals males showed less reactivity than females (*p* = 0.0103) (Fig. 3b).

In contrast, we also found significant differences in the proliferation EdU assay when comparing sociosexual groups (F_(2, 53)_ = 12.81, *p* < 0.0001) but not between sex (F_(1, 53)_ = 0.8781, *p* = 0.3530). For females, only the FeSCM group presented a significant increase in the percentage of EdU + /Nestin + cells compared to the FeCo (*p* = 0.0298). In contrast, for males, an increase was observed between MaSE and MaCo (*p* = 0.0090) and between MaSCM and MaCo (*p* = 0.0014) (Fig. 3d).

### Differentiation potential of SVZ-derived neurosphere cells

Other subgroups of attached neurospheres at D15 were divided into six and ten additional days of culture (D21 and D25, respectively) without growth factors to assess their differentiation potential (experimental timeline in Fig. 1). To identify neuroblasts, doublecortin (DCX) was detected by immunostaining and the relative migration of cells surrounding the neurospheres was measured at D21. The presence of DCX suggests that neurosphere-derived cells are neuroblasts or migrating cells (Fig. 4a). DCX immunoreactivity data between the control and sociosexual groups demonstrated statistically significant differences in females (F _(2, 20)_ = 18.57, *p* < 0.0001) and males (F _(2, 20)_ = 14.57, *p* = 0.0001). In females, the relative intensity of DCX + increased in cultures derived from FeSCM compared to that in the control group (*p* < 0.0001), while in males, the DCX signal increased in the MaSE group (*p* = 0.0004) compared with its respective control group (Fig. 4b). Significant differences were found in relative migration between social groups (F _(2, 30)_ = 24.34, *p* < 0.0001) and between sexes (F _(1, 30)_ = 33.69, p < 0.0001). Post hoc tests demonstrated an increase in the migration distance in FeSe (*p* = 0.005) and FeSCM (*p* < 0.0001) in comparison to FeCo, although in males, there were no changes between the groups (Fig. 4c). Comparison between the sexes demonstrated no significant differences between control groups, while in the social groups the migration distance was higher in females than males (FeSE vs. MaSE, *p* < 0.0272; FeSCM vs. MaSCM, *p* < 0.0001) (Fig. 4c).

To identify differences in differentiation potential, another group of attached neurospheres was cultured for ten days without adding mitotic factors (experimental timeline in Fig. 1). Mature neuronal and glial phenotypes were determined using anti-MAP2 and anti-GFAP antibodies, respectively (Fig. 5a). The female groups showed statistically significant differences in MAP2 immunoreactivity (F _(2, 27)_ = 21.36, *p* < 0.0001). Post hoc tests demonstrated an increase in FeSE (*p* < 0.0001) and FeSCM (*p* < 0.0001) compared to the control group (Fig. 5b). Similarly, significant differences in MAP2 were observed in male voles (F _(2, 26)_ = 7.071, *p* = 0.0035). Thus, MaSE (*p* = 0.0101) and MaSCM (*p* = 0.0077) showed an increase in comparison to MaCo (Fig. 5b). GFAP immunoreactivity data showed significant differences in female voles (F _(2, 26)_ = 37.54, *p* < 0.0001). Also, we detected a decrease in GFAP in FeSE (*p* < 0.0001) and FeSCM (*p* < 0.0001) compared to that in FeCo (Fig. 5c).

Finally, significant differences in GFAP were found in male voles (F _(2, 26)_ = 43.37, *p* < 0.0001), where MaSE (*p* < 0.0001) and MaSCM (*p* < 0.0001) decreased in comparison with MaCo (Fig. 5c).

### Sociosexual conditions modified the outcome of growth factors and hormones on the number and growth of SVZ-derived neurospheres

FGF2 and EGF are conventional molecules that maintain the in vitro proliferative and neurogenic potential of NSCs isolated from the adult/fetal central nervous system in both mice and humans [40, 43–45]. As a first approach, we determined the effect of EGF (20 ng/mL) or FGF2 (20 ng/mL) on SVZ-derived neurospheres. At D12, we quantified the total number and growth of Co (FeCo, MaCo) and SCM (FeSCM, MaSCM) voles after daily administration of the molecules or without growth factors (WoGF) (Fig. 6).

**Fig. 7:**
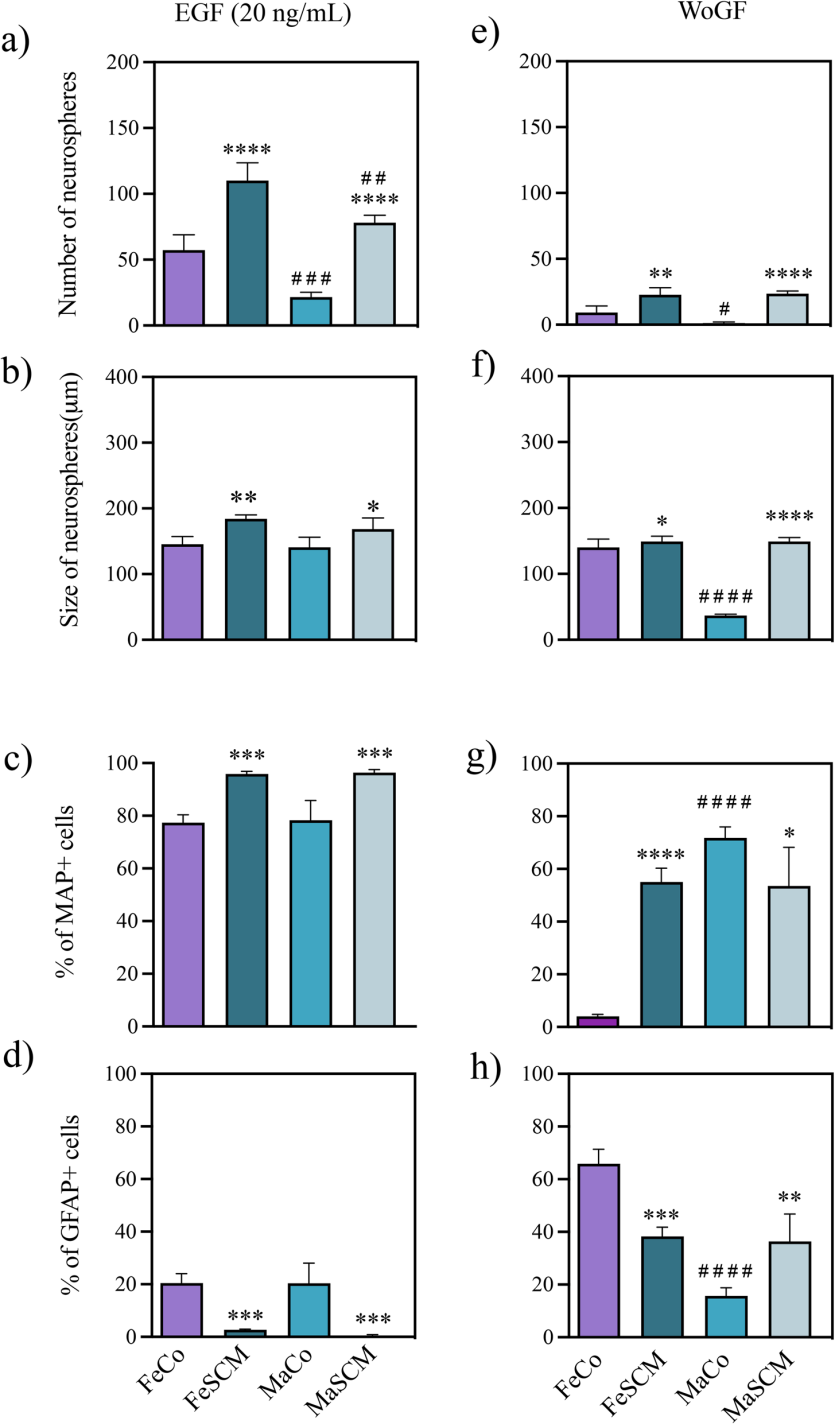
Epidermal Growth Factor (EGF) promotes the formation of neurospheres and neurogenesis from SVZ-isolated cells in the adult prairie vole. Number (**a**) and size (**b**) of SVZ-isolated neurospheres formed under EGF (20 ng/mL) treatment for twelve days in female (Fe) and male (Ma) voles either control (Co) or with social cohabitation and mating (SCM) stimulus. Number of MAP2 + (**c**) and GFAP + (**d**) cells with EGF (20 ng/mL) in neurospheres cultured for fifteen days under adherent conditions. Number (**e**) and size (**f**) of neurospheres cultured without growth factors (WoGF) twelve days after the start of culture. Number of MAP2 + (**g**) and GFAP + (**h**) cells WoGF in neurospheres cultured for fifteen days under adherent conditions. Data were analyzed with a two-way ANOVA followed by Tukey’s post hoc test. n = 4 subjects per group. Each SCM group was compared with its respective Control group in both sexes (FeCo vs FeSCM, MaCo vs MaSCM), and females were compared with male in each sociosexual condition (FeCo vs MaCo, FeSCM vs MaSCM). SCM different from Co in the same sex, *p < 0.05, **p < 0.01, ***p < 0.001, ****p < 0.0001. Female different from male in the same sociosexual condition, ^#^p < 0.05, ^##^p < 0.01, ^###^p < 0.001, ^####^p < 0.0001

There were differences in the number of neurospheres after EGF treatment due to both the sociosexual factor (F_(1, 12)_ = 130.8, *p* < 0.0001; FeCo vs. FeSCM, *p* < 0.0001; MaCo vs. MaSCM, *p* < 0.0001) and sex (F_(1, 12)_ = 50.52, *p* < 0.0001; FeCo vs. MaCo, *p* < 0.0009; FeSCM vs. MaSCM, *p* < 0.0023), although no significant differences in sociosexual and sex interactions were found (F_(1, 12)_ = 0.1613, *p* = 0.6950) (Fig. 7a and Additional file 1: Table S1). When measuring the diameter of the neurospheres on day 12, there were differences between SCM voles and their respective controls (F _(1, 12)_ = 25.38, *p* = 0.0003; FeCo vs. FeSCM, *p* = 0.0066; MaCo vs. MaSCM, *p* = 0.0478), but without differences between sexes (FeCo vs. MaCo, *p* = 0.9537; FeSCM vs. MaSCM, *p* = 0.3898) (Fig. 7b and Additional file 1: Table S1). Interestingly, in SCM voles, there was greater neurosphere formation even without the addition of any growth factor (WoGF) than in their control counterparts (F_(1, 12)_ = 90.37, *p* < 0.0001; FeCo vs. FeSCM, *p* = 0.0014; MaCo vs. MaSCM, *p* < 0.0001); while comparing between sexes, there were only differences in the control animals (FeCo vs. MaCo, *p* = 0.040) (Fig. 7e and Additional file 1: Table S2). In WoGF group, comparisons of diameter measurements performed similarly to neurosphere number counts, with differences between the sociosexual groups (F _(1, 12)_ = 346.9, *p* < 0.0001; FeCo vs FeSCM, *p* = 0.0312; MaCo vs MaSCM, *p* < 0.0001), while when comparing between sexes, there were only differences between control group (FeCo vs FeSCM, *p* < 0.0001) (Fig. 7f and Additional file 1: Table S2). The number of neurospheres was almost null with FGF2 treatment (like WoGF); therefore, this condition was not included in the subsequent treatments, which indicated that this molecule does not promote SVZ-derived neurosphere formation in adult voles (data not shown).

The neurospheres previously derived under WoGF or EGF conditions were attached to promote their outgrowth and subsequent differentiation (for the EGF condition, treatment continued every third day). Fifteen days later, the number of neurosphere-derived MAP2 + and GFAP + cells were analyzed (Fig. 6). However, outgrowth was almost null in Co-WoGF animals and the cell density differed between the control and SCM groups (data not shown). Therefore, unlike previous experiments where MAP2 or GFAP immunoreactivity was analyzed by relative density, we quantified the percentage of MAP2 + or GFAP + cells in relation to the DAPI cells.

EGF-only treatment recapitulated the EGF- and FGF2-supplemented cultures effects, with higher percentages of MAP2 + cells and lower percentages of GFPA + cells in the SCM groups compared to their respective controls (F_(1, 12)_ = 78.71, *p* < 0.0001; FeCo vs. FeSCM, *p* = 0.0002; MaCo vs. MaSCM, *p* = 0.0002 for MAP2, while that F_(1, 12)_ = 79.51, *p* < 0.0001; FeCo vs. FeSCM, *p* = 0.0004; MaCo vs. MaSCM, *p* = 0.001 for GFAP) (Fig. 7c and d and Additional file 1: Table S1) (Additional file 1: Figure S1). However, in the WoGF condition, FeSMC showed a higher and a lower number of MAP2 + and GFAP + , respectively, as compared to their control counterparts (*p* < 0.0001 for MAP2 and *p* = 0.0003 for GFAP) (Fig. 7g, h and Additional file 1: Table S2), whereas it decreased the MAP2 percentage (*p* = 0.0339, Fig. 7g) and increased GFAP percentage *(p* = 0.0030, Fig. 7h*)* in MaSCM as compared with Co group (Additional file 1: Figure S2).

Interestingly, although we did not find any difference between the sexes (F_(1, 12)_ = 0.1112, *p* = 0.7445 for MAP2; F_(1, 12)_ = 0.3261, *p* = 0.5785 for GFAP) in the EGF condition, in WoGF condition there was a difference between female and male controls (F_(1, 12)_ = 67.62, *p* < 0.0001 for MAP2; F_(1, 12)_ = 67.31, *p* < 0.0001 for GFAP), with a higher percentage of MAP2 + cells (*p* < 0.0001) and a lower percentage of GFAP + cells (*p* < 0.0001) in MaCo when compared to FeCo (Fig. 7g, h and Additional file 1: Table S2). These data suggest that without the addition of external factors to the media, there is a sexual dimorphism in proliferation and differentiation of SVZ-cells in vitro in controls animals. However, in SCM stimuli, this phenomenon disappears. On the other hand, when EGF is added to the cultures, the difference between female and male controls vanishes because both respond to EGF to promote proliferation (diameter of neurospheres) and the preference for neural differentiation over glial lineage in all the groups (control and SCM).

**Fig. 8:**
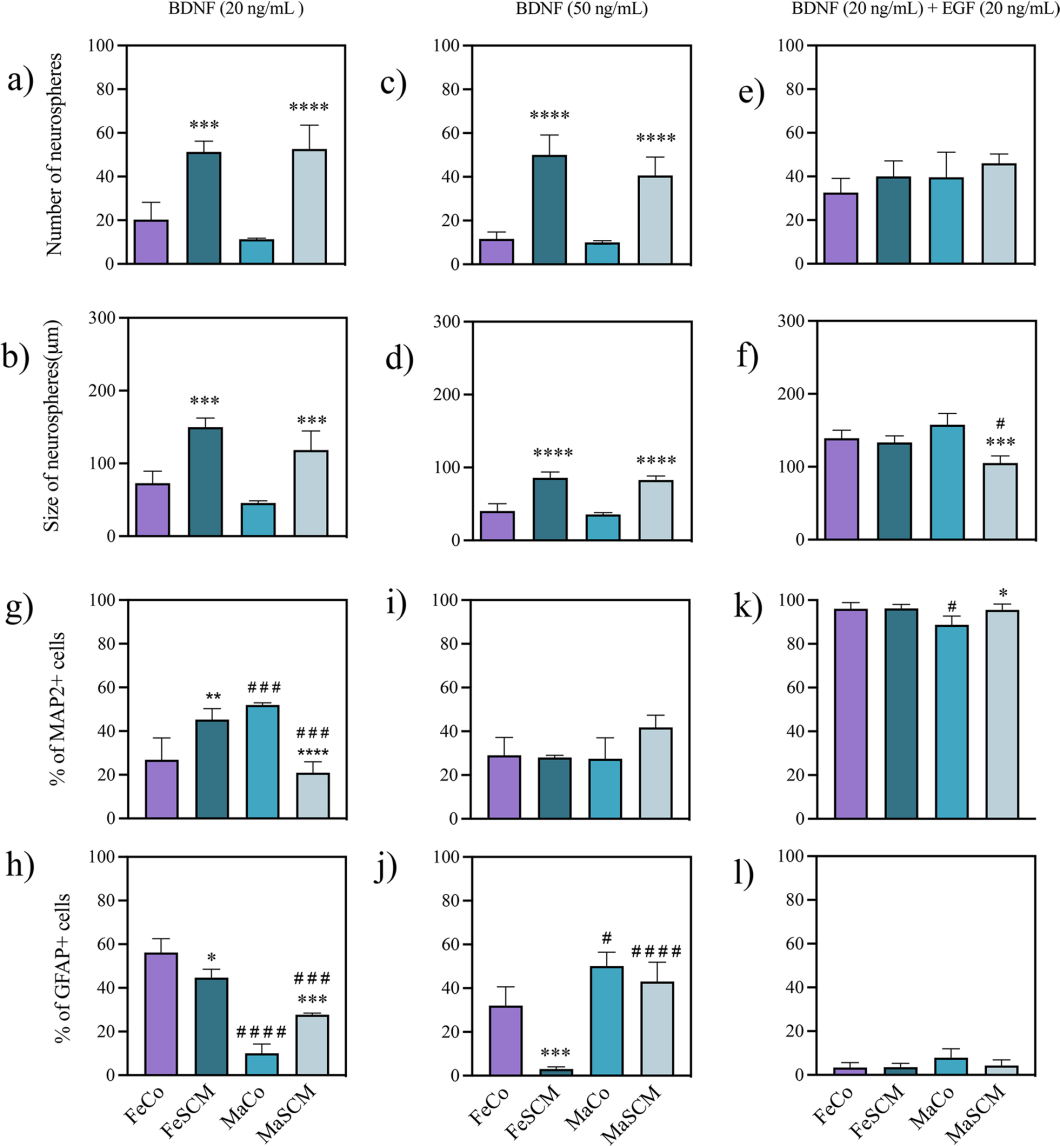
Effect of brain derived neurotrophic factor (BDNF) in the formation of neurospheres and neurogenesis from SVZ-isolated cells in the adult prairie vole. Number of neurospheres formed (**a, c, e**) and their diameter (**b, d, f**) twelve days after the start of culture with BDNF treatments. Percentage of MAP2 + (**g**, **i**, **k**) and GFAP + (**h, j, l**) cells in neurospheres’s cell outgrowth cultured for fifteen days under adherent conditions with BDNF treatments. BDNF treatments: 20 ng/mL, 50 ng/mL and 20 ng/mL BDNF with EGF (20 ng/mL) co-treatment. Data of were analyzed with a two-way ANOVA followed by Tukey’s post hoc test. n = 4 subjects per group of each sex. SCM different from Co in the same sex, *p < 0.05, **p < 0.01, ***p < 0.001, ****p < 0.0001. Female different from male in the same sociosexual condition, ^#^p < 0.05, ^###^p < 0.001, ^####^p < 0.0001

**Fig. 9:**
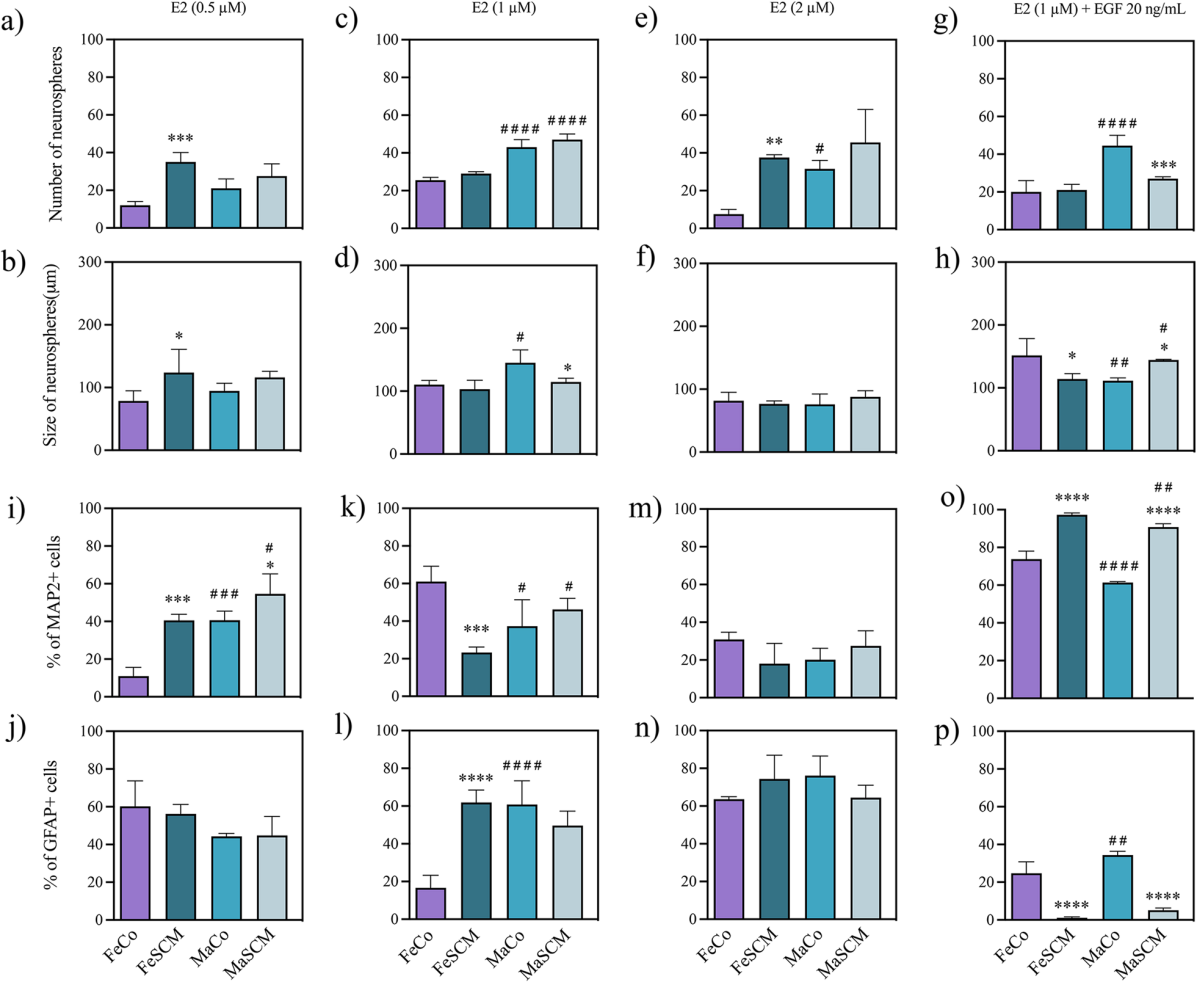
Effect of estradiol (E2) in the formation of neurospheres and neurogenesis from SVZ-isolated cells in the adult prairie vole. Number of neurospheres formed (**a, c, e, g**) and their diameter (**b, d, f, h**) twelve days after the start of culture with E2 treatments. Percentage of MAP2 + (**i, k, m, o**) and GFAP + (**j, l, n, p**) cells in neurospheres’s cell outgrowth cultured for fifteen days under adherent conditions. E2 treatments: 0.5 μM, 1 μM, 2 μM and 1 μM with EGF (20 ng/mL) co-treatment. Data were analyzed with a two-way ANOVA followed by Tukey’s post hoc test. n = 4 subjects per group of each sex. SCM different from Co in the same sex, *p < 0.05, **p < 0.01, ***p < 0.001, ****p < 0.0001. Female different from male in the same sociosexual condition, ^#^p < 0.05, ^##^p < 0.01, ^###^p < 0.001, ^####^p < 0.0001

**Fig. 10:**
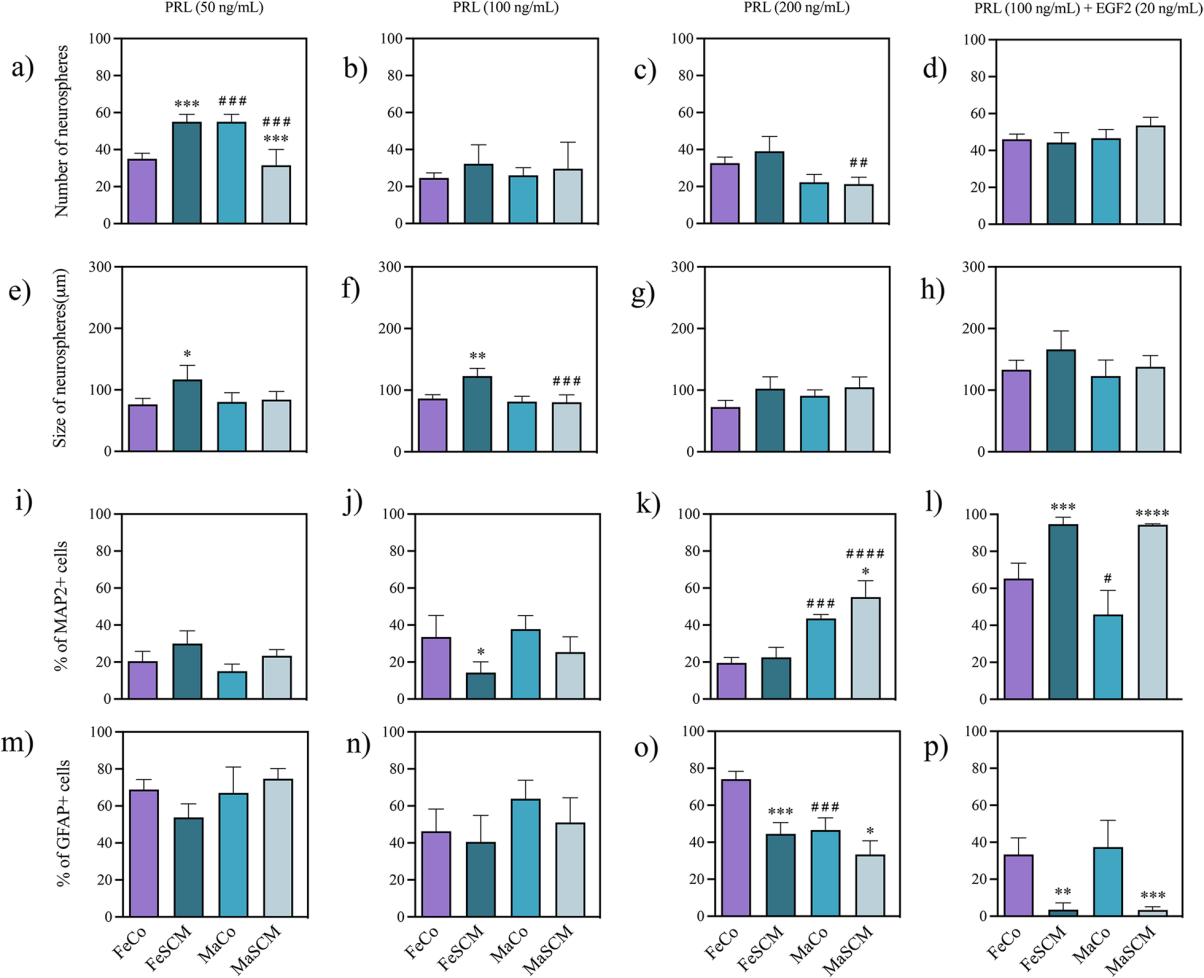
Effect of prolactin (PRL) in the formation of neurospheres and neurogenesis from SVZ-isolated cells in the adult prairie vole. Number of neurospheres formed (**a**–**d**) and their diameter (**e**–**h**) twelve days after the start of culture with PRL treatments. Percentage of MAP2 + (**i**–**l**) and GFAP + (**m**–**p**) cells in neurospheres cultured for fifteen days under adherent conditions. PRL treatments: 50 ng/mL, 100 ng/mL, 200 ng/mL and 100 ng/mL with EGF (20 ng/mL) co-treatment. Data were analyzed with a two-way ANOVA followed by Tukey’s post hoc test. n = 4 subjects per group of each sex. SCM different from Co in the same sex, *p < 0.05, **p < 0.01, ***p < 0.001, ****p < 0.0001. Female different from male in the same sociosexual condition, ^#^p < 0.05, ^##^p < 0.01, ^###^p < 0.001, ^####^p < 0.0001

**Fig. 11:**
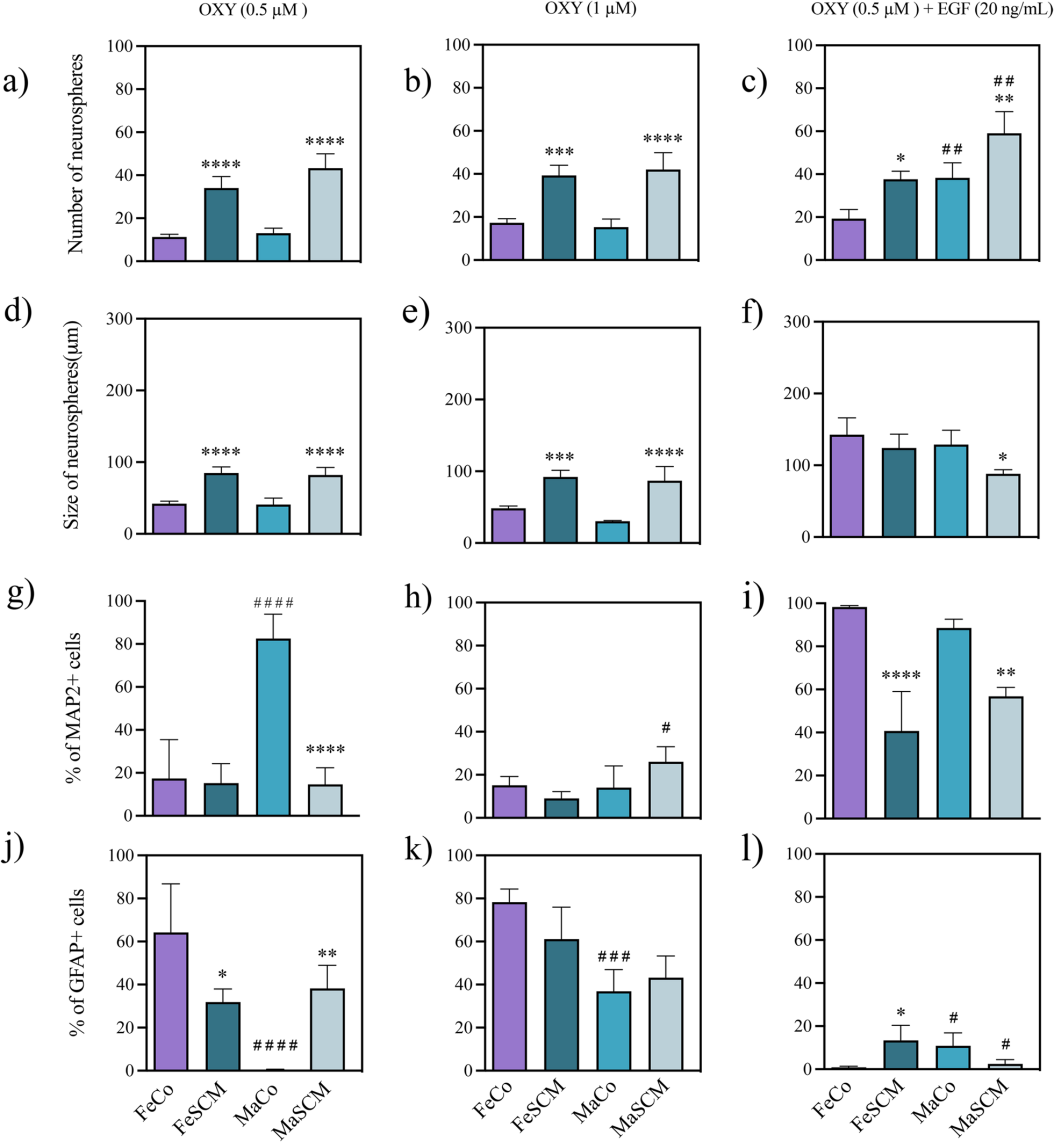
Effect of oxytocin (OXY) in the formation of neurospheres and neurogenesis from SVZ-isolated cells in the adult prairie vole. Number of neurospheres formed (**a**–**c**) and their diameter (**d**–**f**) twelve days after the start of culture with OXY treatments. Percentage of MAP2 + (**g**–**i**) and GFAP + (**j**–**l**) cells in neurospheres cultured for fifteen days under adherent conditions. OXY treatments: 0.5 μM, 1 μM, and 1 μM with EGF (20 ng/mL) co-treatment. Data were analyzed with a two-way ANOVA followed by Tukey’s post hoc test. n = 4 subjects per group of each sex. SCM different from Co in the same sex, *p < 0.05, **p < 0.01, ***p < 0.001, ****p < 0.0001. Female different from male in the same sociosexual condition, ^#^p < 0.05, ^##^p < 0.01, ^###^p < 0.001, ^####^p < 0.0001

**Fig. 12:**
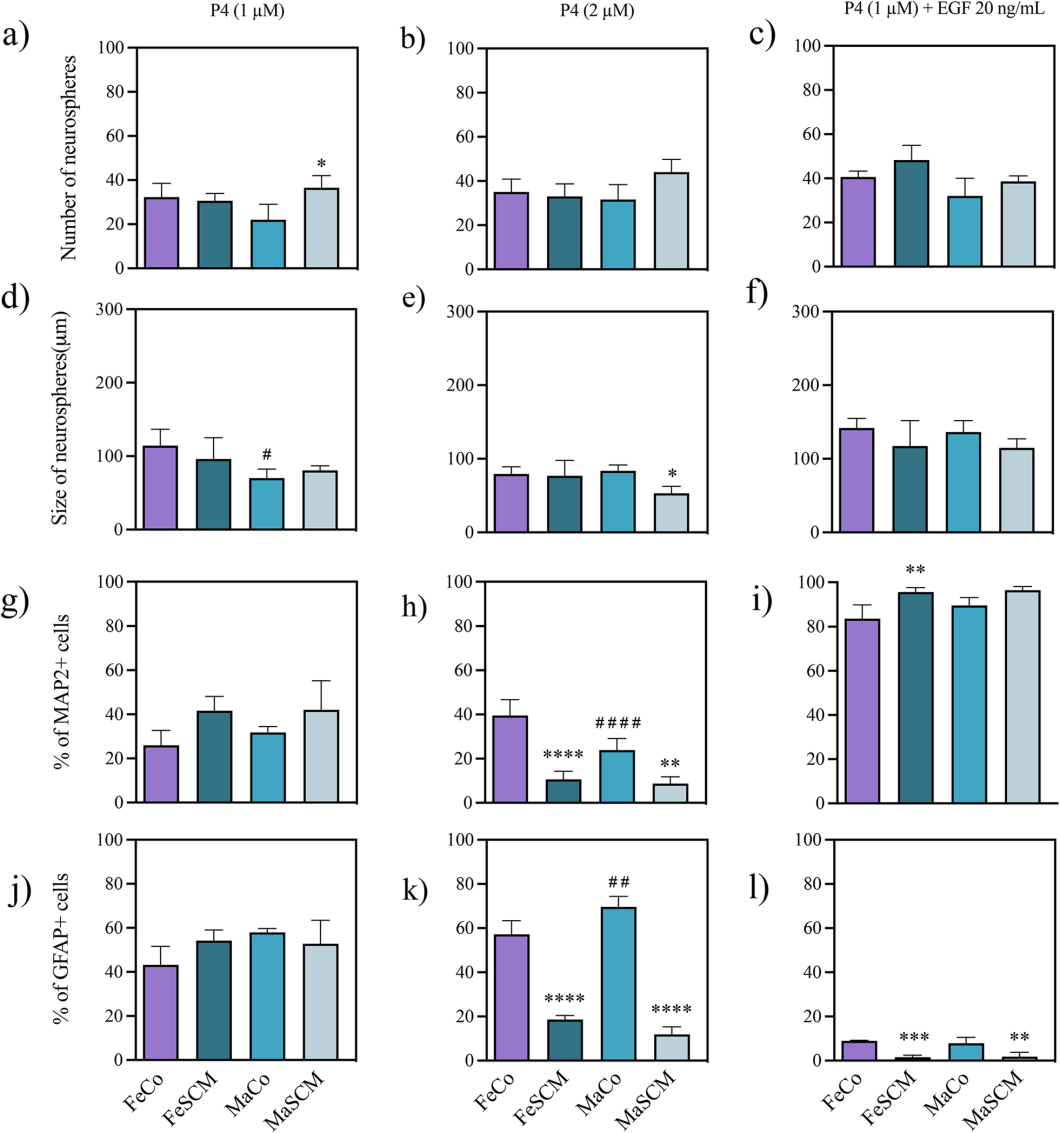
Effect of progesterone (P4) in the formation of neurospheres and neurogenesis from SVZ-isolated cells in the adult prairie vole. Number of neurospheres formed (**a**–**c**) and their diameter (**d**–**f**) twelve days after the start of culture with P4 treatments. Percentage of MAP2 + (**g**–**i**) and GFAP + (**j**–**l**) cells in neurospheres cultured for fifteen days under adherent conditions. P4 treatments: 1 μM, 2 μM, and 1 μM with EGF (20 ng/mL) co-treatment. Data were analyzed with a one-way ANOVA followed by Tukey’s post hoc test. n = 4 subjects per group of each sex. SCM different from Co in the same sex, *p < 0.05, **p < 0.01, ***p < 0.001, ****p < 0.0001. Female different from male in the same sociosexual condition, ^#^p < 0.05, ^##^p < 0.01, ^####^p < 0.0001

In addition to EGF, several growth factors and hormones regulate the proliferation and birth of new neurons in vivo and in vitro isolated from neurogenic niches in mammals, which are controlled by behavioral changes, sociosexual stimuli, stress, stroke injury, lactation, pregnancy, among others (see the review for steroid hormones [46, 47], prolactin [48, 49], brain-derived neurotrophic factor [50], progesterone [51] and oxytocin [52]). Next, we evaluated the formation and differentiation of SVZ-derived neurospheres under single treatments with brain-derived neurotrophic (BDNF) (50, 100, and 200 ng/mL) (Fig. 8), estradiol (E2) (0.5, 1, and 2 µM) (Fig. 9), prolactin (PRL) (50, 100, and 200 ng/mL) (Fig. 10), oxytocin (OXY) (1 and 2 µM) (Fig. 11) and progesterone (P4) (1 and 2 µM) (Fig. 12). Additionally, a group co-administered EGF (20 ng/mL) was included for each growth factor or hormone (PRL, 100 ng/mL; BDNF, 50 ng/mL; P4, 1 μM; E2, 1 μM; and OXY, 0.5 μM). We relied on previous studies that used specific concentrations of factors in cultured neural cells [53–57]. At D15, neurospheres were cultured on Matrigel to adhere to and promote differentiation. In this step, the supplementation of molecules was continued every third day (experimental timeline in Fig. 6).

### BNDF treatments

#### Proliferation

We found significant differences between the sociosexual groups in the number and growth of SVZ-derived neurospheres, with 20 ng/mL (F _(1, 12)_ = 101.7, p < 0.0001 for number; F_(1, 12)_ = 78.99, *p* < 0.0001 for growth) and 50 ng/mL concentrations (F _(1, 12)_ = 116.9, *p* < 0.0001 for number; F_(1, 12)_ = 173.5, *p* < 0.0001 for growth), whereas BDNF 20 ng/mL plus EGF (20 ng/mL) induced a significant difference only in neurosphere size (F _(1, 12)_ = 25.36, *p* = 0.0003) (Additional file 1: Table S3). When comparing the SCM groups with their respective control group, the number and growth of SVZ-derived neurospheres were higher in each sex with BDNF 20 ng/mL (*p* = 0.003 and* p* < 0.0001 for number; *p* = 0.0002 and *p* = 0.0003 for females and males, respectively to growth) (Fig. 8a, b) and BDNF 50 ng/mL (females: for number *p* < 0.0001 and growth *p* < 0.0001; males: for number *p* < 0.0001 and growth *p* < 0.0001) (Fig. 8c, d). These effects were not observed when EGF was added to the culture; we only found a decrease in the size of the neurospheres in the MaSCM as compared with the MaCo group (*p* = 0.0002) (Fig. 8f).

When comparing between sexes, there were no differences in both the formation and size of neurospheres with BDNF treatment. Only the interaction with the sociosexual factor at 20 ng/mL plus EGF (20 ng/mL) (F_(1, 12)_ = 16.41, *p* = 0.0016) showed a decrease size in MaSCM as compared with FeSCM (*p* = 0.0213) (Fig. 8f).

#### Differentiation

When we compared among sociosexual groups, there was a significant interaction between both factors (sex and sociosexual) with 20 ng/mL (F_(1, 12)_ = 64.77, *p* < 0.0001 for MAP2 and F_(1, 12)_ = 46.51, *p* < 0.0001 for GFAP), 50 ng/mL (F_(1, 12)_ = 4.954, *p* = 0.0460 for MAP2 and F_(1, 12)_ = 9.921, *p* = 0.0084 for GFAP) and 20 ng/mL plus 20 ng/mL EGF (F_(1, 12)_ = 5.132, *p* = 0.0428 for MAP2) (Additional file 1: Figure S3 and Table S3). Indeed, the 20 ng/ml BDNF concentration increased (*p* = 0.0053) and decreased (*p* < 0.0001) the percentage of MAP2 + cells in females and males, respectively, as compared with the Co group (Fig. 8g). On the other hand, GFAP + percentage decreased and increased in females (*p* = 0.017) and males (*p* = 0.0004), respectively, at the 20 ng/mL dose (Fig. 8h), whereas a decrease in the percentage of GFAP + cells was observed only in females (*p* = 0.0004) with the 50 ng/mL treatment as compared with Co groups (Fig. 8j). When BDNF treatment (20 ng/mL) was supplemented with EGF (20 ng/mL), there was only an increase in the percentage of MAP2 + cells in the SCM compared to its Co counterpart (*p* = 0.0301) (Fig. 8k).

We observed differences attributed to the sex factor, for MAP2 it was only under the 20 ng/mL plus EGF treatment (F_(1, 12)_ = 7.810, *p* = 0.0162), whereas in the GFAP data, there were differences in the 20 ng/mL (F _(1, 12)_ = 219.0, *p* < 0.0001) and 50 ng/mL (F_(1, 12)_ = 70.01, *p* < 0.0001) treatments (Additional file 1: Table S3). We found significant interactions between sex and sociosexual factors with 20 ng/mL (F_(1, 12)_ = 64.77, *p* < 0.0001 for MAP2; F_(1, 12)_ = 46.51, *p* < 0.0001 for GFAP) and 50 ng/mL (F_(1, 12)_ = 4.954, *p* = 0.0460 for MAP2; F_(1, 12)_ = 9.921, *p* = 0.0084 for GFAP) and 20 ng/mL plus EGF (F _(1, 12)_ = 5.132, *p* = 0.0428 for MAP2) (Additional file 1: Table S3). When comparing between sexes under treatment with 20 ng/mL BDNF, in MaCo there was a higher percentage of MAP2 + cells (*p* = 0.0004) and a lower percentage of GFAP + cells (*p* < 0.0001) than in FeCo, but in SCM animals, females had higher percentages of both markers than males (*p* = 0.0006 for MAP2, *p* = 0.0005 for GFAP) (Fig. 8g, h). At a concentration of 50 ng/mL BDNF, there were no sex differences in the percentages of neuronal cells, but both Co and SCM males had a higher percentage of glial cells than females (MaCo vs. FeCo, *p* = 0.0142; MaSCM vs. FeSCM, *p* < 0.0001) (Fig. 8i, j). Finally, when adding EGF (20 ng/mL) plus 20 ng/mL BDNF, the percentages were very similar, except in the neuronal lineage, with a significant decrease in MaCo compared to their female counterparts (*p* = 0.0173) (Fig. 8k) (Additional file 1: Figure S3).

### Estradiol treatments

#### Proliferation

We found significant differences between the sociosexual groups in the neurospheres number and growth, with 0.5 μM E2 (F_(1, 12)_ = 36.17, *p* < 0.0001 for number; F_(1, 12)_ = 9.613, *p* = 0.0092 for growth) and 1 μM E2 (F_(1, 12)_ = 7.965, *p* = 0.0154 for number; F_(1, 12)_ = 8.352, *p* = 0.0136 for growth), whereas 2 μM E2 (F_(1,12)_ = 23.12, *p* = 0.0004 for number) and 1 μM E2 plus EGF (F_(1, 12)_ = 14.28, *p* = 0.0026 for number) had effects only on the number as compared with the Co groups (Additional file 1: Table S4). The 0.5 μM concentration increased the number and size of neurospheres in the FeSCM compared to those in the Co group (*p* < 0.0001 and *p* = 0.0499, respectively) (Fig. 9a, b), whereas we only observed a decrease in the size (1 μM) in the MaSCM (*p* = 0.0287, Fig. 9d) and an increase (2 μM) in the number of neurospheres in the FeSCM (*p* = 0.0028, Fig. 9e) as compared with the control groups. Remarkably, we found a decrease in the size of neurospheres (*p* = 0.0138, Fig. 9h) in FeSCM and a diminished number (*p* = 0.0005, Fig. 9g) and increased neurosphere size (*p* = 0.0289, Fig. 9h) in MaSCM with 1 μM E2 and EGF co-treatment.

Regarding sex differences, there were changes in both parameters with 1 μM (F_(1, 12)_ = 178.4, *p* < 0.0001 for number; F_(1, 12)_ = 12.27, *p* = 0.0044 for growth), but only in number with both 2 μM (F_(1, 12)_ = 12.23, *p* = 0.0044) and 1 μM plus EGF (F_(1, 12)_ = 48.80, *p* < 0.0001) (Additional file 1: Table S4). Additionally, there was an interaction between sex and sociosexual factors only with the addition of EGF for both parameters (F_(1, 12)_ = 17.95, *p* = 0.0012 for number; F_(1, 12)_ = 24.44, *p* = 0.0003 for growth) (Additional file 1: Table S4). Intriguingly, with 1 μM, there was a greater number of neurospheres in each group of males relative to their female counterparts (*p* < 0.0001 for both MaCo vs. FeCo and MaSCM vs. FeSCM), although only increased in size when compared between control animals (MaCo vs. FeCo, *p* = 0.0134), while there was no difference in the SCMs (Fig. 9c, d). Similarly, in both the 2 µM and 1 µM plus EGF (20 ng/mL) treatments, we found a higher number of neurospheres in MaCo vs. FeCo (*p* = 0.0137 and *p* < 0.0001, respectively) (Fig. 9e–g). Finally, when 1 µM plus EGF (20 ng/mL) was added to the culture, the size of the neurospheres was larger in FeCo than in MaCo (*p* = 0.0084), but in FeSCM, there was a decrease with respect to MaSCM (*p* = 0.0471) (Fig. 9h).

#### Differentiation

We found also significant differences attributed to the sociosexual factor in MAP2 + cells percentage with 0.5 μM (F_(1, 12)_ = 45.14, *p* < 0.0001), 1 μM (F _(1, 12)_ = 10.78, *p* = 0.0065) and 1 μM E2 plus EGF (F_(1, 12)_ = 507.3, *p* < 0.0001); while for GFAP + cells there were differences with 1 μM (F _(1, 12)_ = 15.19, *p* = 0.0021) and 1 μM E2 plus EGF (F _(1, 12)_ = 261.2, *p* < 0.0001) treatments (Additional file 1: Table S4). Indeed, we observed an increment in the MAP2 + cells in each sex for SCM with the 0.5 μM (*p* = 0.0002 and *p* = 0.0429 for females and males, respectively) (Fig. 9i) and 1 μM plus EGF (*p* < 0.0001 for both, Fig. 9o) conditions, while 1 μM decreased MAP2 + percentage (*p* = 0.0003, Fig. 9k) and increased GFAP + percentage (*p* < 0.0001, Fig. 9l) in FeSCM as compared with the Co group. Lastly, we found a decrease in the GFAP percentage in each sex for SCM (*p* < 0.0001 for both) with 1 μM E2 plus EGF co-treatment with respect to their control groups (Fig. 9p).

When we compared between sexes, there were differences in the percentage of MAP2 cells under the 0.5 μM (F_(1, 12)_ = 45.62, *p* < 0.0001) and 1 μM plus EGF (20 ng/mL) (F_(1, 12)_ = 65.01, *p* < 0.0001) E2 treatments; whereas in GFAP, there were differences under 1 μM (F_(1, 12)_ = 13.32, *p* = 0.0033) and 1 μM plus EGF (20 ng/mL) (F_(1, 12)_ = 17.33, *p* = 0.0013) E2 treatments (Additional file 1: Table S4). Remarkably, with 1 μM treatment that there was a significant interaction between both factors (sex and sociosexual) in both MAP2 (F_(1, 12)_ = 28.15, *p* = 0.0002) and GFAP (F_(1, 12)_ = 41.64, *p* < 0.0001) scores. We found a higher percentage of MAP2 + in males than in females under treatment with 0.5 μM in both sociosexual groups (Co and SCM, *p* = 0.0002 and *p* = 0.0410, respectively) (Fig. 9i) and with 1 μM only in SCM males, there was a higher percentage of MAP2 + cells as compared with FeSCM (*p* = 0.0143) (Fig. 9k). However, we found a decrease in MAP2 percentage in MaCo vs FeCo at 1 μM (*p* = 0.0113) (Fig. 9k), whereas in 1 μM plus EGF regimen, there was a decrease in males relative to females in both Co and SCM groups (*p* < 0.0001 and *p* = 0.0097, respectively) (Fig. 9o). Finally, differences in the percentage of GFAP were found in treatments with 1 μM (*p* < 0.0001) and 1 μM plus EGF (*p* = 0.0062) with a greater increase in MaCo than in FeCo (Fig. 9l and p) (Additional file 1: Figure S4).

### Prolactin treatments

#### Proliferation

With the 50 and 100 ng/mL PRL treatments, there were differences between sociosexual groups with respect to neurosphere size (F_(1, 12)_ = 7.731 and *p* = 0.0166, F _(1,12)_ = 12.37, *p* = 0.0042, respectively), whereas with the lowest concentration of the hormone (50 ng/mL), only the interaction between the factors of sex and sociosexual group was significant in the number of neurospheres (F_(1, 12)_ = 66.83, p < 0.0001) (Additional file 1: Table S5). Indeed, larger neurospheres were found in these PRL FeSCM groups (*p* = 0.0167 in 50 ng/mL and *p* = 0.0013 in 100 ng/mL treatment) than in the FeCo groups (Fig. 10e and f), whereas PRL (50 ng/mL) treatment increased the number of neurospheres in each sex of the SCM groups (*p* = 0.0009 for females and *p* = 0.0002 for males) as compared with Co conditions (Fig. 10a).

Sex differences were found in the 200 ng/mL treatment for the number of neurospheres (F_(1, 12)_ = 29.38, *p* = 0.0002), while with 100 ng/mL, there were differences with respect to size (F_(1, 12)_ = 22.13, *p* = 0.0005) (Additional file 1: Table S5). Thus, it was found that at 50 ng/mL in Co Ma, there was a greater number of neurospheres than in their female counterparts (p = 0.0009) (Fig. 10a), whereas in SCM males, there was a reduction in both number (by 50 and 200 ng/mL, p = 0.0002 and p = 0.0019, respectively) (Fig. 10a and c) and size (p = 0.0003 for 100 ng/mL, Fig. 10f) with respect to FeSCMs.

#### Differentiation

PRL treatment showed significant differences in the percentage of cells differentiated into MAP2 or GFAP, 100 ng/mL (F _(1, 12)_ = 13.81, *p* = 0.0029 for MAP2), 200 ng/mL (F _(1, 12)_ = 7.086 and *p* < 0.0207 for MAP2; F _(1, 12)_ = 48.05 and *p* < 0.0001 for GFAP), and 100 ng/mL plus EGF 20 ng/mL (F_(1, 12)_ = 96.286 and *p* < 0.0001 for MAP2; F _(1, 12)_ = 52.97 and *p* < 0.0001 for GFAP) (Additional file 1: Table S5). We found a decrease (*p* = 0.0333, Fig. 10j) in the MAP2 + percentage in FeSCM with 100 ng/mL PRL compared with the control. In contrast, an increase (*p* = 0.0483, Fig. 10k) in MAP2 percentage was observed in MaSCM with 200 ng/mL PRL and both SCM sex groups treated with PRL plus EGF co-administration increased MAP2 percentage (*p* = 0.0010 for females and *p* < 0.0001 for males; Fig. 10l). In addition, a decrease in the percentage of GFAP + cells was found in each sex in SCM groups with 200 ng/mL (*p* = 0.0001 for females and *p* = 0.0454 for males, Fig. 10o) and 100 ng/mL PRL with EGF doses (*p* = 0.00215 for females and *p* = 0.0007 for males, Fig. 10p) as compared with its control group.

On the other hand, when comparing between sexes, the results showed differences at concentrations of 200 ng/mL PRL for both neural markers (F_(1, 12)_ = 107.3, *p* < 0.0001 for MAP2; F_(1, 12)_ = 39.20, *p* < 0.0001 for GFAP) and PRL plus EGF treatment only for the neuronal lineage (F_(1, 12)_ = 6.208, *p* = 0.0284) (Additional file 1: Table S5). Furthermore, the sex-sociosexual interaction was significant after treatment with 100 ng/mL PRL plus EGF for MAP2 (F_(1, 12)_ = 5.726, p = 0.0340) and with 200 ng for GFAP (F_(1, 12)_ = 7.049, *p* = 0.0210). Thus, with 200 ng/mL PRL, both Co and SCM males had a higher percentage of MAP2 + cells than females (p = 0.0002 and p < 0.0001, respectively) (Fig. 10k) and we found a lower percentage of GFAP + cells in MaCo as compared with FeCo (p = 0.0002, Fig. 10o). Finally, when EGF was added in conjunction with PRL, the percentage of MAP2 was lower in males than in females only in the control animals (p = 0.0215, Fig. 10l) (Additional file 1: Figure S5).

### Oxytocin treatments

#### Proliferation

OXY treatments exhibited between sociosexual groups significantly differences in the number and size of neurospheres with 0.5 μM (F_(1, 12)_ = 141.8, *p* < 0.0001 for number; F _(1, 12)_ = 99.30, p < 0.0001 for growth); 1 μM (F_(1, 12)_ = 94.16, *p* < 0.0001 for number; F_(1, 12)_ = 82.09, *p* < 0.0001 for growth) and 0.5 μM plus EGF (F_(1, 12)_ = 33.21, *p* < 0.0001 for number; F_(1,12)_ = 10.26; *p* = 0.0076 for growth) (Additional file 1: Table S6). Interestingly, all treatments with OXY increased the number (*p* < 0.0001 in 0.5 μM for both sexes; *p* = 0.0002 and *p* < 0.0001 in 1 μM for females and males, respectively; *p* = 0.0145 and *p* = 0.0059 in 1 μM plus EGF for females and males, respectively) (Fig. 11a–c) and size of neurospheres in SCM groups (*p* < 0.0001 in 0.5 μM to both sex; *p* = 0.0006 and *p* < 0.0001 in 1 μM to female and male, respectively; *p* = 0.0382 in 1 μM plus EGF to male) (Fig. 11d–f). In contrast, only the 1 μM OXY plus EGF (20 ng/mL) condition there was a sexual dimorphism in the number of neurospheres (F_(1, 12)_ = 35.64, *p* < 0.0001), males having higher values than their female counterparts under both sociosexual conditions (p = 0.0112 for Co; p = 0.0045 for SCM) (Fig. 11c).

#### Differentiation

When we compared the potential of the neurospheres to differentiate with the OXY treatments, we found significant differences at 0.5 μM (F _(1, 12)_ = 32.92, *p* < 0.0001) and 0.5 μM OXY plus EGF (F _(1, 12)_ = 87.83 and *p* < 0.0001) for MAP2 (Additional file 1: Table S6) with the sociosexual factor. In contrast, for GFAP there was only significance in the interaction of the two factors (sex and sociosexual) in all OXY treatments (F_(1, 12)_ = 29.68, *p* = 0.0001 for 0.5 μM; F_(1, 12)_ = 4.836, *p* = 0.0482 for 1 μM; F_(1, 12)_ = 19.70, *p* = 0.0008 for 0.5 μM plus EGF) (Additional file 1: Table S6). The MAP2 + cells percentage diminished with 0.5 μM (*p* < 0.0001) in MaSCM (Fig. 11g), whereas a decrease also was observed in SCM groups (*p* < 0.0001 and *p* = 0.0021 to female and male, respectively) treated with EGF co-treatment as compared with Co groups (Fig. 11i). In addition, with the lowest hormone concentration, the GFAP + cell percentage diminished in the FeSCM (*p* = 0.0184) and increased (*p* = 0.0062) in the MaSCM group compared to the Co groups (Fig. 11j). Finally, EGF co-administration increased (*p* = 0.0130) the GFAP + cell percentage in FeSCM compared with the control group (Fig. 11l).

When identifying differences between sexes, there were significances at 0.5 μM (F_(1, 12)_ = 28.01, *p* = 0.0002 for MAP2; F_(1, 12)_ = 19.96, *p* = 0.0008 for GFAP) and 1 μM (F_(1, 12)_ = 5.775, *p* = 0.0333 for MAP2; F_(1, 12)_ = 30.69, *p* = 0.0001 for GFAP) OXY for both neural markers (Additional file 1: Table S6). Interestingly, MAP2 percentages were higher in males than females at concentrations of 0.5 μM (FeCo vs MaCo, p < 0.0001, Fig. 11g) and 1 μM (FeSCM vs MaSCM, p = 0.0162, Fig. 11h), while with GFAP the percentages were lower for 0.5 μM and 1 μM between Co groups (*p* < 0.0001 and p = 0.0007, respectively) (Fig. 11j and k). When EGF was added to the 0.5 OXY treatment, there was a higher percentage of GFAP in MaCo than in FeCo (p = 0.0471). In SCM, the results were contrary, with FeSCM higher than MaSCM (p = 0.0294) (Fig. 11l) (Additional file 1: Figure S6).

### Progesterone treatments

#### Proliferation

Finally, the number and growth of SVZ-derived neurospheres were also different between the experimental sociosexual groups when the cultures were treated with 1 μM (F _(1, 12)_ = 4.931, *p* = 0.0464 for number) and 2 μM (F _(1, 12)_ = 6.273, *p* = 0.0277 for growth) (Additional file 1: Table S7). We found only an increase (*p* = 0.0163, Fig. 12a) in neurospheres number with 1 μM P4 in MaSCM compared to the Co group, whereas a decrease in the size of neurospheres was observed in the MaSCM group compared to the Co group (2 μM, *p* = 0.0292, Fig. 12e).

Only the 1 µM treatment had a sex difference in neurosphere size (F_(1, 12)_ = 9.401, *p* = 0.0098), with a greater number of spheres in females than in MaCo (p = 0.0324) (Fig. 12d), whereas there was no significant interaction between sex and sociosexual factor in any of the P4 treatments.

#### Differentiation

We also found a significant sociosexual difference in the differentiation potential towards both neural phenotypes with 2 μM (F _(1, 12)_ = 76.90, *p* < 0.0001 for MAP2; F _(1, 12)_ = 488.0, *p* < 0.0001 for GFAP) and 1 μM P4 plus EGF (F _(1, 12)_ = 24.05, *p* = 0.0004 for MAP2; F _(1, 12)_ = 61.31 and *p* < 0.0001 for GFAP) treatments (Additional file 1: Table S7). Intriguingly, treatment with 2 μM P4 decreased the percentage of MAP2 and GFAP + cells in both FeSCM (*p* < 0.0001 for MAP2 and GFAP) and MaSCM (*p* = 0.0051 for MAP2 and *p* < 0.0001 for GFAP) as compared with the Co group (Fig. 12h and k), while the co-treatment with EGF increased (*p* = 0.0040, Fig. 12i) the MAP2 + percentage in FeSCM and decreased (*p* = 0.0003 and *p* = 0.0016 for females and males, respectively) the percentage of GFAP + cells in each sex in SCM as compared with the control group (Fig. 12l). In addition, with the 2 μM concentration, there was a significant difference between sexes in the percentage of MAP2 + cells (F_(1, 12)_ = 12.26, *p* = 0.0044) and with the interaction with the sociosexual factor (F _(1, 12)_ = 7.419, p = 0.0185), while for GFAP, only differences were reported with the interaction (F_(1, 12)_ = 19.34, *p* = 0.0009) (Additional file 1: Table S7). Thus, there was a lower percentage of MAP2 + cells (*p* = 0.0051, Fig. 12h) and a higher percentage of GFAP + cells (*p* = 0.0079, Fig. 12k) in control males than in control females with 2 μM of the hormone (Additional file 1: Figure S8).

## Discussion

We observed phenotypic differences in neurospheres derived from isolated cells of the SVZ of the prairie vole based on their socio-sexual status (Co, SE, SCM) and sex (Fe, Ma). First, the number and size of neurospheres were greater in SCM and SE animals than in their respective controls (Fig. 2b, c). Additionally, there was a greater number of neurospheres in females compared to males, regardless of their social-sexual status (Fig. 2b). Conventionally, in neurosphere assays, the number of neurospheres formed in a primary culture is related to the initial number of NSCs [58, 59]. Hence, the greater number of neurospheres in the SCM and SE animals suggests that there were more self-renewing NSCs in these groups. Furthermore, females had a higher initial number of NSCs than males. The size of the neurospheres also correlates with the proliferation or amplification potential of NSCs and neural progenitors [35, 58, 59]. Although the proliferation of neurospheres increased in SCM and SE animals compared to controls, this effect was lost when comparing between sexes (Fig. 3).

In terms of undifferentiated cell identity, we used Nestin, a widely used marker of NPCs, to detect the proportion of progenitor cells isolated from the multipotent SVZ of prairie voles that had sociosexual experiences. The results indicated a higher Nestin immunoreactive signal in neurospheres from the FeSCM and MaSE groups, suggesting an increase in the proportion of NPCs in these groups (Fig. 3a, b). The SE and SCM groups also presented an increase in the percentage of Edu + /Nestin + cells, indicating a greater proliferative capacity (Fig. 3c, d). These findings suggest that social exposure that facilitates social bonding also induces plastic changes such as proliferation in the SVZ neurogenic niche.

In female social groups (FeSE and FeSCM), we observed variations in cellular migration through DCX detection and the measurement of neurosphere outgrowth (Fig. 4). Although SE males showed differences in DCX immunoreactivity (Fig. 4b), there were no differences in the migratory distance between the MaCo, MaSE, and MaSCM groups (Fig. 4c). Additionally, our analysis of the comparison between the sexes revealed that FeSE and FeSCM exhibited an increase in migration with respect to their male counterparts (Fig. 4b). We previously reported a protocol for neurosphere formation from neurogenic niches for this species, as well as decreased DCX in neurosphere-derived cells from males relative to females, suggesting an intrinsic sex-dependent difference [33]. Interestingly, an increase in differentiation towards the neuronal lineage was observed in neurosphere-derived cells from all social experimental groups, in contrast to the control groups (Fig. 5). These findings suggest that social exposure promotes the commitment of undifferentiated cells from the SVZ to a specific lineage different from that of neurogenic niches from sexually naive animals.

Accordingly, our group recently reported an increase in the number of NPCs in the dorsal region of the SVZ of male voles that experienced SE or SCM [31]. In adult mice, NPCs from the dorsal and ventral SVZ generate neuroblasts that migrate to the rostral migratory stream to differentiate into the OB and incorporate tyrosine hydroxylase +, calbindin + , or calretinin + periglomerular interneurons [15, 60]. We suggest that neurogenesis in the SVZ and new neurons in the OB contributes to the formation of long-term memory, which is necessary for pair-bonding consolidation [31]. Female SVZ-isolated cells from the SCM group displayed an increase in proliferation, demonstrated by the results of immunoreactive nestin (Fig. 3b), the percentage of double EdU/nestin + cells (Fig. 3d), and immunoreactivity to DCX, compared to the control group (Fig. 4b). However, while we observed differences in the EdU proliferation assay in the MaSCM group (Fig. 3d), there was an increase in immunoreactivity to nestin (Fig. 3b), double EdU/nestin + cells (Fig. 3d), and DCX (Fig. 4b) in the maSE group. These findings suggest that social exposure without mating is sufficient to induce the proliferation of SVZ-isolated cells in males, in contrast to that in females.

Thus, olfactory sensory input in both male and female voles has the potential to influence the differentiation fate of SVZ progenitor cells that integrate into the OB. However, female voles require sexual stimulation, as opposed to male voles, to increase the proliferation of undifferentiated SVZ cells. These changes in neural plasticity could play a role in determining socio-sexual behavior in voles. For example, female voles exposed to sexually active males without mating do not exhibit partner preference or pair bonding [6]. However, if 6 h of cohabitation includes mating, females form a pair bond, suggesting that the release of mating-induced hormones, such as dopamine, vasopressin, and oxytocin, could promote changes in plastic processes, such as proliferation. Additionally, while NPCs from the SVZ give rise to neuronal, gliogenic, and oligodendrocytic phenotypes, the mechanisms of the neurogenic niche environment that involves NPCs to generate mostly neurons or the three lineages of the central nervous system are unknown. Nonetheless, under in vitro conditions with high concentrations of growth factors, NPCs display a multipotent potential, with a high rate of differentiation towards glial lineage and without a predominance of neuronal lineage, as occurs in vivo [15].

In accordance with the literature, we observed the presence of glial lineage in our neurosphere cultures derived from control groups. Intriguingly, SVZ-derived neurosphere cultures from SE or SCM voles had a higher rate of neuronal lineage (MAP2 +) and fewer glial cells (GFAP +) as compared to the control group (Fig. 5). This might be due to the delayed differentiation process towards glial cells in both the SE and SCM groups. This postponement allows the cells to direct their differentiation towards the neuronal lineage upon exposure to social cues and copulation. Interestingly, this commitment to neuronal lineage is maintained even after in vitro culture, mirroring the control group that exhibted glial differentiation. To determine the role of EGF and FGF2 in neurosphere formation, we cultured SVZ-isolated cells from both SCM and control voles under the following conditions: without growth factors, with EGF (20 ng/mL), or with FGF2 (20 ng/mL). Although few neurospheres were formed with FGF2 (data not shown) or without any treatment, EGF was sufficient to form neurospheres (Fig. 7a). EGF treatment also recapitulated the previous experiments under standard conditions, with an increase in the percentage of MAP2 + cells and a decrease in GFAP + cells in SCM compared to control groups (Fig. 7c, d and Additional file 1: Figure S1). This suggests that EGF promotes the proliferation of SVZ-isolated cells with a preferential fate towards the neuronal lineage in the adult prairie vole.

In cultures without growth factors (WoGF) from SCM voles, SVZ-isolated cells formed neurospheres (Fig. 7e). However, in FeSCM-WoGF, the tendency to differentiate towards neurons continued, while in MaSCM-WoGF, it decreased leading to an increase in the percentage of MAP2- and GFAP-positive cells, respectively (Fig. 7). This implies that there are initial EGF-independent signals acting on the SVZ cells in the brain of SCM voles. These signals persist under in vitro conditions, endowing them with a different potential for proliferation and differentiation. The plasticity of neural progenitors in differentiating into unexpected cell types has been documented in other rodent models. For example, undifferentiated SVZ cells from the adult mouse brain are destined for dopaminergic or GABAergic interneuron differentiation, which then integrates into the OB. However, when SVZ tissue is grafted ectopically in a non-proliferative region (striatum), the surviving cells differentiate into non-neuronal lineages expressing glial (S100, GFAP, and Vimentin) and oligodendrocyte (Olig2 and CNPase) markers [61]. Additionally, in the absence of extrinsic factors (no EGF addition), there were sex-dependent differences in all parameters evaluated, but only in the controls, so that sociosexual stimuli diminished the differences between sexes. Curiously, with EGF treatment, sex-attributed differences disappeared, and only sociosexual differences remained (except for the initial number of neurospheres). To investigate whether other factors may be involved in the observed sociosexual and sex-dependent differences, we conducted further experiments.

We generated neurospheres from SVZ-isolated cells in control (FeCo, MaCo) and SCM (FeSCM, MaSCM) voles and treated them with several molecules (BDNF, E2, PRL, OXY, and P4) for 15 days. We then allowed the neurospheres to differentiate under adherent conditions for another 15 days with supplemented growth factors or hormones every third day (Fig. 6). In FeSCM-BDNF (20 ng/mL), the number and size of neurospheres were higher compared to those in FeCo. Furthermore, a higher percentage of MAP2 + cells and a lower percentage of GFAP + cells were observed (Fig. 8a, b, g, h). In males, both 20 ng/mL and 50 ng/mL BDNF significantly increased the number and growth of neurospheres in MaSCM compared to MaCo (Fig. 8a–d). However, at 20 ng/mL BNDF, there was a lower percentage of MAP2 + cells and a higher percentage of GFAP + cells (Fig. 8g, h). Notably, BDNF (20 ng/mL) and EGF (20 ng/mL) did not promote differentiation into neuronal identity in either group (SCM or Co) for both sexes, resulting in a minimal number of glial cells (Fig. 8k, l). This suggests that BDNF can mimic the effect of EGF in both female and male voles but is insufficient to increase neurogenesis in the MaSCM. In contrast, the simultaneous administration of BDNF and EGF synergistically enhanced the neurogenic potential in control animals, similar to SCM (Fig. 8k). These effect of BDNF are consistent with previous findings in other rodents.

Previous studies have demonstrated that the administration of BDNF in combination with FGF2 and EGF increases proliferation and neurogenesis in the SVZ of adult rats [62]. Additionally, it has been shown that 40 ng/mL of BDNF promotes proliferation and neuronal differentiation in neurospheres derived from hippocampus-isolated cells of the same species [63]. However, when comparing the effects of multiple factors with BDNF supplementation, it appears that proliferation parameters are influenced by sociosexual factors, with sex differences becoming evident only at the differentiation stage. To investigate the effects of estrogen on proliferation in the SVZ, as reported in rats [64], different concentrations of E2 were used. While E2 (0.5 μM) and E2 (2 μM) maintained a greater number of neurospheres, a specific dose of 1 μM had the opposite effect. In FeSCM, there was a lower percentage of MAP2 + cells and a higher percentage of GFAP + cells compared to FeCo. Conversely, in MaSCM, there was no significant difference in all measurements under 0.5, 1, and 2 E2 μM treatments, except for the percentage of MAP2 + cells. However, when E2 (1 μM) was combined with EGF (20 ng/mL), there was no increase in the number and growth of neurospheres obtained from the SCM groups compared with their control counterparts. The percentages of MAP2 and GFAP were similar to those in the standard conditions, indicating higher neurogenesis and lower gliogenesis in the SCM compared to the Co for both sexes (Fig. 9).

While it is well-established that the sexual behavior of male and female prairie voles is influenced by the integration of newborn neurons into the OB [65], the effects of estrogen on cell proliferation in the OB of female prairie voles depending on the species and region [66]. Therefore, it was not unexpected that we observed differential effect of estrogen on the formation and differentiation of SVZ-derived neurospheres between sexes and sociosexual groups. Indeed, our study revealed that the impact of estrogen on neurogenesis was influenced by sociosexual factors and the interaction between sex and this factor. For instance, we noted that the negative effect of 1 μM estrogen on neurogenesis was specific to the female control group (Additional file 1: Figure S4).

We observed that PRL promoted an increase in the size of FeSCM neurosphere at concentrations of 50 and 100 ng/mL (Fig. 10e, f). Interestingly, even the lowest concentration of PRL increased the number of neurospheres in the MaSCM group when compared to the MaCo group (Fig. 10a). These findings suggest that the regulation of cell proliferation in SVZ-isolated cells is influenced by the hormone´s concentration, yielding varying results across sociosexual and sex groups, regardless of differentiation stage. Notably, at a concentration of 100 ng/mL, PRL decreased the percentage of MAP2 + cells in females (Fig. 10j), while at 200 ng/mL, it increased this percentage in males (Fig. 10k) within the SCM groups. These results indicate that PRL may decreases neurogenesis in the FeSCM, but at higher concentrations, it might promote neurogenesis, at least in MaSCM voles, sex-specific effects. Co-administration with EGF rescued these outcomes, leading to increased neurogenesis and reduced gliogenesis in SCM compared to Control group (Fig. 10l). This support EGF as a PRL-independent dominant pathway for promoting differentiation towards neurogenesis.

OXY stimulates proliferation and neurogenesis in the dentate gyrus of the hippocampus in rats [67, 68], and it increases the size of neurospheres formed from the rat hypothalamic neuronal cell line H32 [54]. Since hormones are to know to play a role in the neurochemical regulation of pair bonding [4, 69], they may also be involved in the molecular mechanisms regulating SVZ neurogenic proliferation and differentiation. Notably, OXY treatments mainly attribute differences in proliferation parameters (number and size) to sociosexual factors, whereas in differentiation (MAP2 + and GFAP + cells), sex is a factor that induces differential results. In contrast, when administered at a concentration of 0.5 μM in MaSCM, the hormone decreased the number of MAP2 + cells (Fig. 11g) and increased the percentage of GFAP + cells (Fig. 11j). Interestingly, co-administration of EGF did not rescue the increased neurogenesis in either SCM sex; instead, it decreased the percentage of MAP2 + cells compared to their control counterparts (Fig. 11i). Indeed, MaSE and MaSCM have fewer BrdU/NeuN cells in the main OB that migrate from the SVZ [32]. Therefore, we hypothesized that OXY serve as a negative regulator of neurogenesis in males. It would be interesting to analyze whether this hormone plays a sex-dependent differential role in in vivo neurogenesis in adult prairie voles.

Treatment with P4 (1, 2, and 1 μM plus EGF) did not result in any significant differences in the number of neurospheres, except for 1 μM, which showed an increase in MaSCM compared to MaCo (Fig. 12a). However, there was a significant decrease in the size of neurospheres treated with P4 (2 μM) in MaSCM (Fig. 12e). Interestingly, P4 at 2 μM induced a selective decrease in the percentage of MAP2 and GFAP + cells in the SCM of both sex (Fig. 12h–k). Co-treatment with EGF rescued the percentage of MAP2 + cells, although it increased in FeSCM compared to that in FeCo (Fig. 12i). Furthermore, there was a decrease in the percentage of GFAP + cells compared to that in control voles (Fig. 7l). Previous studies have demonstrated that P4 derivatives, such as dihydroprogesterone and tetrahydroprogesterone, drastically decrease the number of BrdU + cells in the ependymal zone (SVZ) of adult male rats [70]. This suggests that P4 regulates neurogenesis or gliogenesis through decreased proliferation of SVZ progenitors but selectively in voles that cohabitate with mating. Additionally, sex was only important for differentiation rather than proliferation.

These results suggest that signals from growth factors, neurotransmitters, and hormones modulate the proliferation and differentiation of SVZ cells in adult prairie voles and that these signals differ depending on the sociosexual context. The current challenge is to elucidate how these cellular and molecular processes are associated with neural plasticity, cognitive changes, and sociosexual behavior. For example, it would be possible to use the CRISPR-Cas9 gene-editing system to disrupt genes that encode hormones selectively or growth factor receptors expressed in the SVZ to test their functional role in neurogenesis in pair bonding voles [71]. In addition, the molecular mechanisms underlying NPCs fate pre-determination in response to the previously mentioned factors, neurotransmitters, or hormones could be epigenetic. Valproic acid, an inhibitor of histone deacetylase, promotes neuronal fate and simultaneously inhibits gliogenic lineage in hippocampal NPCs [72]. Epigenetic changes in various physiological processes, including those within the central nervous system, in response to previously mentioned factors such as serotonin or estradiol, have been widely documented [73, 74]. Therefore, it is possible to identify signals that are released into or produced within the SVZ niche, which induces a biased preference for neuronal differentiation in NPCs.

### Perspectives and significance

The findings of this study provide valuable insights into the impact of social interactions, growth factors, and hormones on the properties of cells isolated from the subventricular zone (SVZ) in voles. This study suggests that sociosexual and sex factors play distinct roles in cell proliferation and differentiation. Although sex influences control animals, the differences between males and females are diminished in groups with social interactions. The addition of epidermal growth factor eliminates the sex differences observed under certain conditions, emphasizing its role in promoting neurosphere growth and proliferation. Moreover, the identification of hormones and growth factors involved in regulating the self-renewal, proliferation, and cell fate properties of neural stem cells in a sociosexual context have important implications, underscoring their significance in modulating neural stem cell properties to understand the molecular mechanisms underlying such sociosexual behaviors as well as the role of neurogenesis and neurogenic niches in these processes. Further in vivo and in vitro experiments are required to unravel the molecular differences between sociosexual stimuli and control subjects in adult prairie voles. Investigating the receptor density for specific neurotransmitters or hormones, employing single-cell transcriptomics, analyzing the epigenetic landscape, and exploring differential signaling pathways will shed light on the intricate mechanisms underlying the effects of social interactions on SVZ cells. This research contributes to a comprehensive understanding of the plasticity of cell proliferation and neurogenesis, providing insights into the establishment, enhancement, and maintenance of pair bond formation.

## Conclusions

Social interactions that promote pair bonding in voles change the properties of cells isolated from the SVZ. Thus, SE or SCM induces a bias in the differentiation potential in both sexes, while SE is sufficient to promote proliferation in SVZ-isolated cells from male brains. In females, proliferation increases when mating is performed.

On the other hand, when there is no treatment with growth factors or hormones, there are differences (both in the proliferation and differentiation stages) not only associated with sociosexual factors but also by sex. However, the sex factor only influenced control animals, whereas in the groups with social interactions (SCM), there were no differences between males and females. We found that the addition of EGF (20 ng/mL) was sufficient to eliminate the sex differences previously observed in WoGF conditions, so that EGF promoted the number and growth of neurospheres (proliferation) in all treatments, but it is important to mention that the differences attributed to the sociosexual factor were still maintained. Regarding the identification of hormones or growth factors that could be involved in regulating the self-renewal, proliferation, and cell fate properties of NSCs in voles in a sociosexual context, the conclusions based on the cell culture experiments of the neurosphere formation assay were as follows:

1. Sociosexual factors are important for the differences in the proliferation potential of isolated cells under culture conditions with BDNF, E2, and OXY treatments.
2. Sex-dependent results were observed with BDNF and E2 treatments at the differentiation stage, while with OXY, there were also sex-dependent differences, but only in control animals.
3. E2 treatments had the most versatile results, with an effect at both stages (proliferation and differentiation) and with attribution to both sociosexual factors and sex. For example, a specific concentration of 1 µM induces FeSCM to decrease its neuronal differentiation potential, in contrast to conditions without any treatment or to conventional conditions with EGF.
4. With PRL, there are also variable results; for example, concentrations of 50 and 100 ng PRL promote no differences in differentiation potential between all groups, "erasing" the differences attributed to the sociosexual factor observed under conventional conditions with EGF or no treatment at all. On the other hand, co-treatment with 200 ng and 100 ng EGF induced no differences in proliferation, but there were changes in differentiation attributed to both factors (sex and sociosexual).
5. Finally, the most drastic changes were observed in the P4 treatments during differentiation and were attributed to the sociosexual factor. For example, with 2 μM P4, there was no neurite formation in the SCM of female and male mice.

The next question is whether the rise in proliferation and neurogenesis of cells from the SVZ are plastic processes essential for establishing, enhancing, maintaining, or accelerating pair bond formation. Further in vivo and in vitro experiments are needed to identify the molecular differences in SVZ cells between socially exposed and control animals, such as receptor density for specific neurotransmitters or hormones, single-cell transcriptomics, determined epigenetic landscape, and differential signaling pathways, considering the novel relevant role of neurogenesis and neurogenic niches in socio-sexual behaviors.

### Supplementary Information

**Additional file 1: Figure S1.** Representative fluorescence microscopy images of MAP2 + (red) and GFAP + (green) cells in SVZ-derived neurospheres cultured with epidermal growth factor (EGF) (20 ng/mL) treatment from control (Co) and social cohabitation with mating (SCM) groups in female (Fe) and male (Ma) adult voles. Nuclei were stained with DAPI (blue). Scale bars = 50 µm. **Figure S2.** Representative fluorescence microscopy images of MAP2 + (red) and GFAP + (green) positive cells in SVZ-derived neurospheres cultured without growth factors (WoGF) from control (Co) and social cohabitation with mating (SCM) groups in female (Fe) and male (Ma) adult voles. Nuclei were stained with DAPI (blue). Scale bars = 50 µm. **Figure S3.** Representative fluorescence microscopy images of MAP2 + (red) and GFAP + (green) cells in SVZ-derived neurospheres cultured with brain-derived neurotrophic factor (BDNF) (20 ng/mL, 50 ng/mL and 20 ng/mL with EGF (20 ng/mL) co-treatment) from control (Co) and social cohabitation with mating (SCM) groups in female (Fe) and male (Ma) adult voles. Nuclei were stained with DAPI (blue). Scale bars = 50 µm. **Figure S4.** Representative fluorescence microscopy images of MAP2 + (red) and GFAP + (green) cells in SVZ-derived neurospheres cultured with estradiol (E2) (0.5 µM, 1 µM, 2 µM and 1 µM with EGF (20 ng/mL) co-treatment) from control (Co) and social cohabitation with mating (SCM) groups in female (Fe) and male (Ma) adult voles. Nuclei were stained with DAPI (blue). Scale bars = 50 µm. **Figure S5.** Representative fluorescence microscopy images of MAP2 + (red) and GFAP + (green) cells in SVZ-derived neurospheres cultured with prolactin (PRL) (50 ng/mL, 100 ng/mL, 200 ng/mL and 100 ng/mL with EGF (20 ng/mL) co-treatment). from control (Co) and social cohabitation with mating (SCM) groups in female (Fe) and male (Ma) adult voles. Nuclei were stained with DAPI (blue). Scale bars = 50 µm. **Figure S6.** Representative fluorescence microscopy images of MAP2 + (red) and GFAP + (green) cells in SVZ-derived neurospheres cultured with oxytocin (OXY) (0.5 μM, 1 μM and 1 μM with EGF (20 ng/mL) co-treatment) from control (Co) and social cohabitation with mating (SCM) groups in female (Fe) and male (Ma) adult voles. Nuclei were stained with DAPI (blue). Scale bars = 50 µm. **Figure S7.** Representative fluorescence microscopy images of MAP2 + (red) and GFAP + (green) cells in SVZ-derived neurospheres cultured with progesterone (P4) (1 μM, 2 μM and 1 μM with EGF (20 ng/mL) co-treatment) from control (Co) and social cohabitation with mating (SCM) groups in female (Fe) and male (Ma) adult voles. Nuclei were stained with DAPI (blue). Scale bars = 50 µm. **Table S1.** 2way ANOVA results (sociosexual and sex as factors) and Tukey’s multiple comparisons tests for epidermal growth factor (EGF) treatment. Comparisons between sociosexual groups: FeCo-FeSCM and MaCo-MaSCM (black). Comparisons between sexes groups: FeCo-MaCo and FeSCM-MaSCM (red). Non-significant comparisons were omitted (“––" as without any significance). **Table S2.** 2way ANOVA results (sociosexual and sex as factors) and Tukey’s multiple comparisons tests for condition without growth factors or any treatments. Comparisons between sociosexual groups: FeCo-FeSCM and MaCo-MaSCM (black). Comparisons between sexes groups: FeCo-MaCo and FeSCM-MaSCM (red). Non-significant comparisons were omitted (“––" as without any significance). **Table S3.** 2way ANOVA results (sociosexual and sex as factors) and Tukey’s multiple comparisons tests for brain-derived neurotrophic factor (BDNF) treatments. Comparisons between sociosexual groups: FeCo-FeSCM and MaCo-MaSCM (black). Comparisons between sexes groups: FeCo-MaCo and FeSCM-MaSCM (red). Non-significant comparisons were omitted (“––" as without any significance). **Table S4.** 2way ANOVA results (sociosexual and sex as factors) and Tukey’s multiple comparisons tests for estradiol (E2) treatments. Comparisons between sociosexual groups: FeCo-FeSCM and MaCo-MaSCM (black). Comparisons between sexes groups: FeCo-MaCo and FeSCM-MaSCM (red). Non-significant comparisons were omitted (“––" as without any significance). **Table S5.** 2way ANOVA results (sociosexual and sex as factors) and Tukey’s multiple comparisons tests for prolactin treatments. Comparisons between sociosexual groups: FeCo-FeSCM and MaCo-MaSCM (black). Comparisons between sexes groups: FeCo-MaCo and FeSCM-MaSCM (red). Non-significant comparisons were omitted (“––" as without any significance). **Table S6.** 2way ANOVA results (sociosexual and sex as factors) and Tukey’s multiple comparisons tests for oxytocin treatments. Comparisons between sociosexual groups: FeCo-FeSCM and MaCo-MaSCM (black). Comparisons between sexes groups: FeCo-MaCo and FeSCM-MaSCM (red). Non-significant comparisons were omitted (“––" as without any significance). **Table S7.** 2way ANOVA results (sociosexual and sex as factors) and Tukey’s multiple comparisons tests for progesterone treatments. Comparisons between sociosexual groups: FeCo-FeSCM and MaCo-MaSCM (black). Comparisons between sexes groups: FeCo-MaCo and FeSCM-MaSCM (red). Non-significant comparisons were omitted (“––" as without any significance).

## Data Availability

Data and materials will be shared.
