# Supplementary material for: Increased proliferation and neuronal fate in prairie vole brain progenitor cells cultured in vitro: effects by social exposure and sexual dimorphism
Source: Biol Sex Differ. 2023 Nov 2;14:77. doi: 10.1186/s13293-023-00563-2 (PMC10623709; doi:10.1186/s13293-023-00563-2)
Supplement: Supplementary file 1 — Additional file 1: Figure S1. Representative fluorescence microscopy images of MAP2 + (red) and GFAP + (green) cells in SVZ-derived neurospheres cultured with epidermal growth factor (EGF) (20 ng/mL) treatment from control (Co) and social cohabitation with mating (SCM) groups in female (Fe) and male (Ma) adult voles. Nuclei were stained with DAPI (blue). Scale bars = 50 µm. Figure S2. Representative fluorescence microscopy images of MAP2 + (red) and GFAP + (green) positive cells in SVZ-derived neurospheres cultured without growth factors (WoGF) from control (Co) and social cohabitation with mating (SCM) groups in female (Fe) and male (Ma) adult voles. Nuclei were stained with DAPI (blue). Scale bars = 50 µm. Figure S3. Representative fluorescence microscopy images of MAP2 + (red) and GFAP + (green) cells in SVZ-derived neurospheres cultured with brain-derived neurotrophic factor (BDNF) (20 ng/mL, 50 ng/mL and 20 ng/mL with EGF (20 ng/mL) co-treatment) from control (Co) and social cohabitation with mating (SCM) groups in female (Fe) and male (Ma) adult voles. Nuclei were stained with DAPI (blue). Scale bars = 50 µm. Figure S4. Representative fluorescence microscopy images of MAP2 + (red) and GFAP + (green) cells in SVZ-derived neurospheres cultured with estradiol (E2) (0.5 µM, 1 µM, 2 µM and 1 µM with EGF (20 ng/mL) co-treatment) from control (Co) and social cohabitation with mating (SCM) groups in female (Fe) and male (Ma) adult voles. Nuclei were stained with DAPI (blue). Scale bars = 50 µm. Figure S5. Representative fluorescence microscopy images of MAP2 + (red) and GFAP + (green) cells in SVZ-derived neurospheres cultured with prolactin (PRL) (50 ng/mL, 100 ng/mL, 200 ng/mL and 100 ng/mL with EGF (20 ng/mL) co-treatment). from control (Co) and social cohabitation with mating (SCM) groups in female (Fe) and male (Ma) adult voles. Nuclei were stained with DAPI (blue). Scale bars = 50 µm. Figure S6. Representative fluorescence microscopy images of MAP2 + (red) and G [file 13293_2023_563_MOESM1_ESM.docx]

**Figure S1.** Representative fluorescence microscopy images of MAP2-(red) and GFAP-(green) positive cells in SVZ-derived neurospheres cultured with epidermal growth factor (EGF) (20 ng/mL) treatment from control (Co) and social cohabitation with mating (SCM) groups in female (Fe) and male (Ma) adult voles. Nuclei were stained with DAPI (blue). Scale bars = 50 µm.


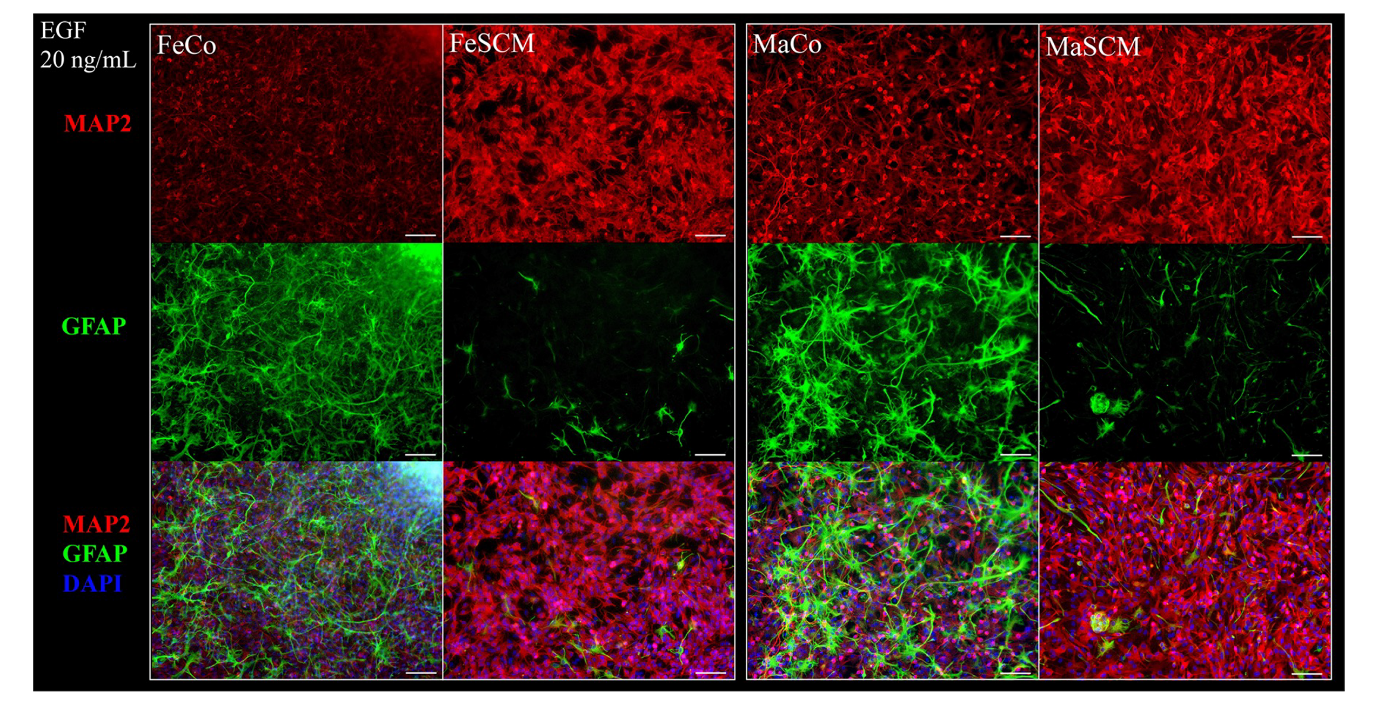


**Figure S2.** Representative fluorescence microscopy images of MAP2-(red) and GFAP-(green) positive cells in SVZ-derived neurospheres cultured without growth factors (WoGF) from control (Co) and social cohabitation with mating (SCM) groups in female (Fe) and male (Ma) adult voles. Nuclei were stained with DAPI (blue). Scale bars = 50 µm.


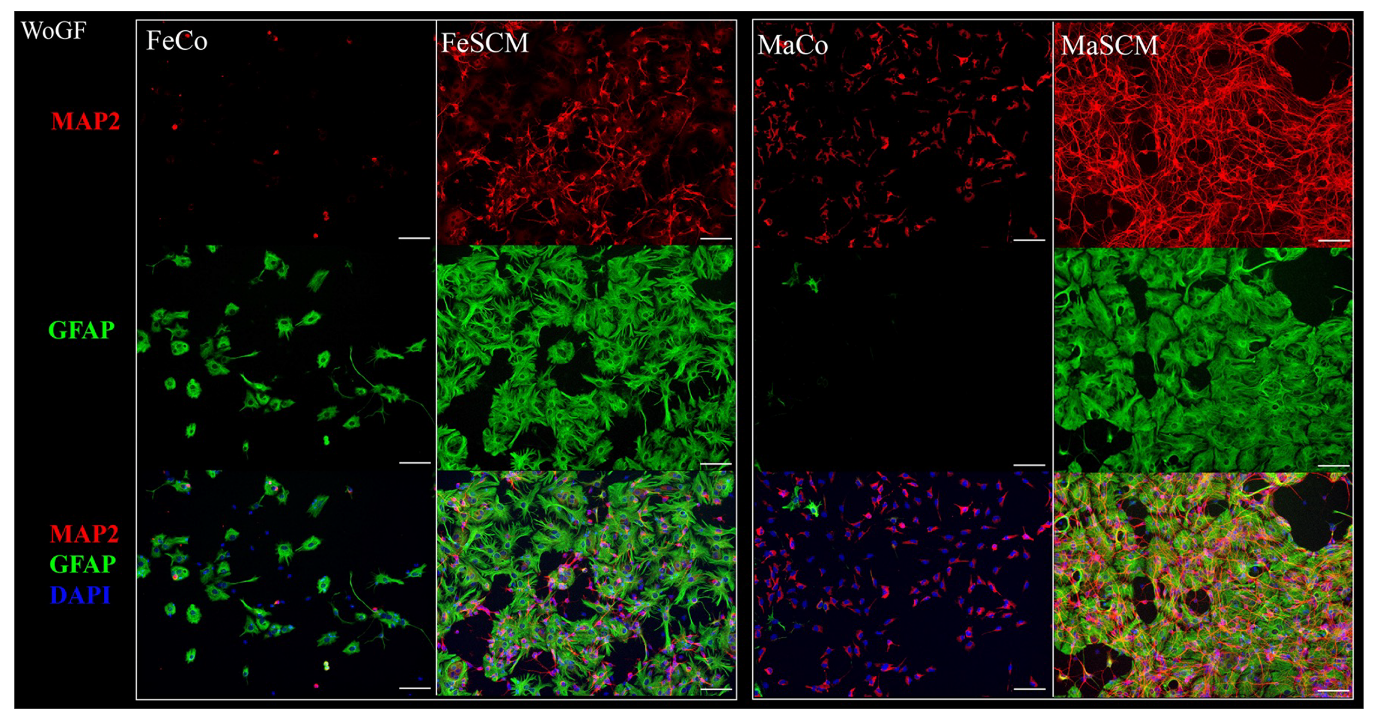


**Figure S3.** Representative fluorescence microscopy images of MAP2-(red) and GFAP-(green) positive cells in SVZ-derived neurospheres cultured with brain-derived neurotrophic factor (BDNF) (20 ng/mL, 50 ng/mL and 20 ng/mL with EGF (20 ng/mL) co-treatment) from control (Co) and social cohabitation with mating (SCM) groups in female (Fe) and male (Ma) adult voles. Nuclei were stained with DAPI (blue). Scale bars = 50 µm.


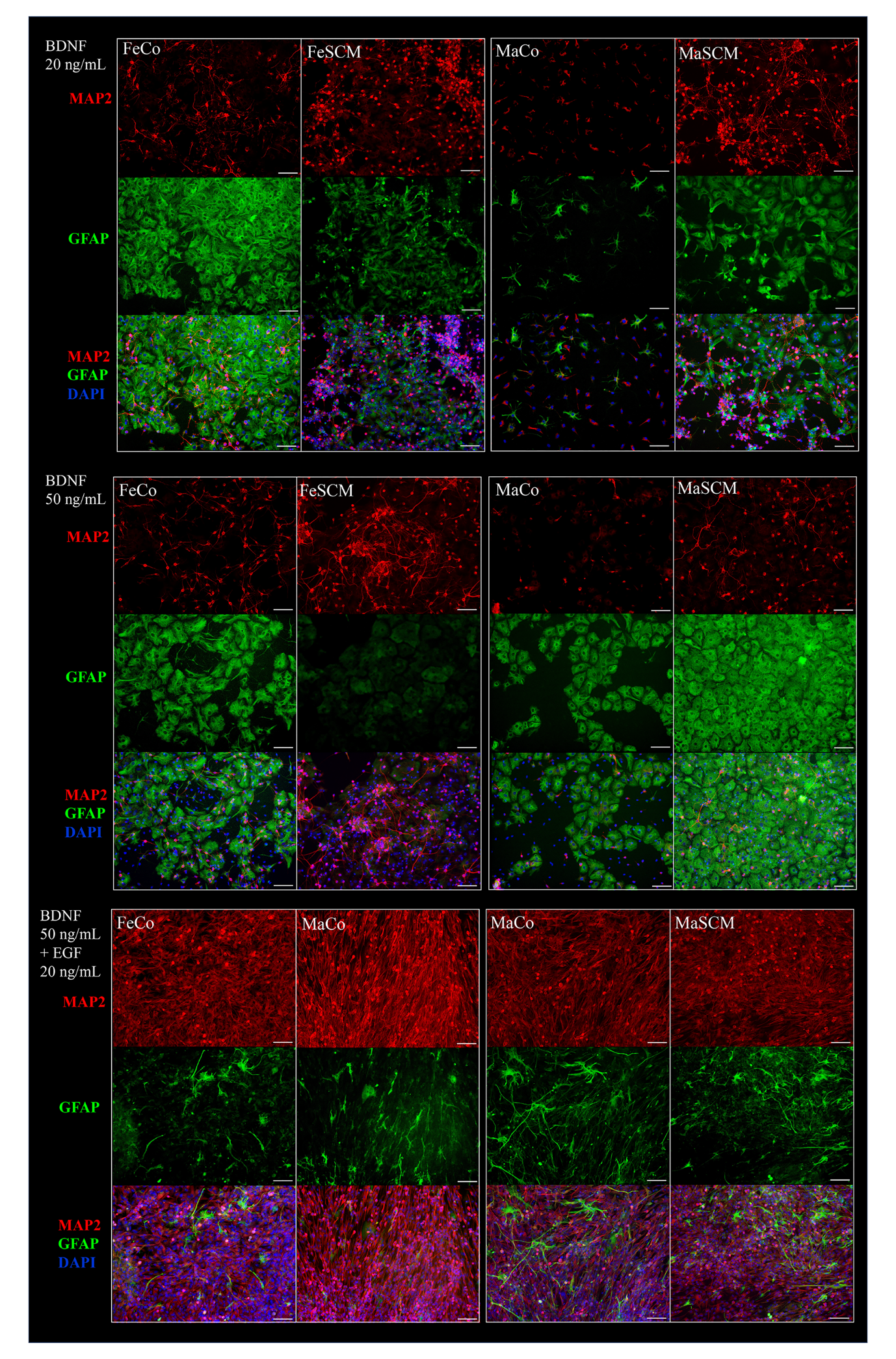


**Figure S4.** Representative fluorescence microscopy images of MAP2-(red) and GFAP-(green) positive cells in SVZ-derived neurospheres cultured with estradiol (E2) (0.5 µM, 1 µM, 2 µM and 1 µM with EGF (20 ng/mL) co-treatment) from control (Co) and social cohabitation with mating (SCM) groups in female (Fe) and male (Ma) adult voles. Nuclei were stained with DAPI (blue). Scale bars = 50 µm.


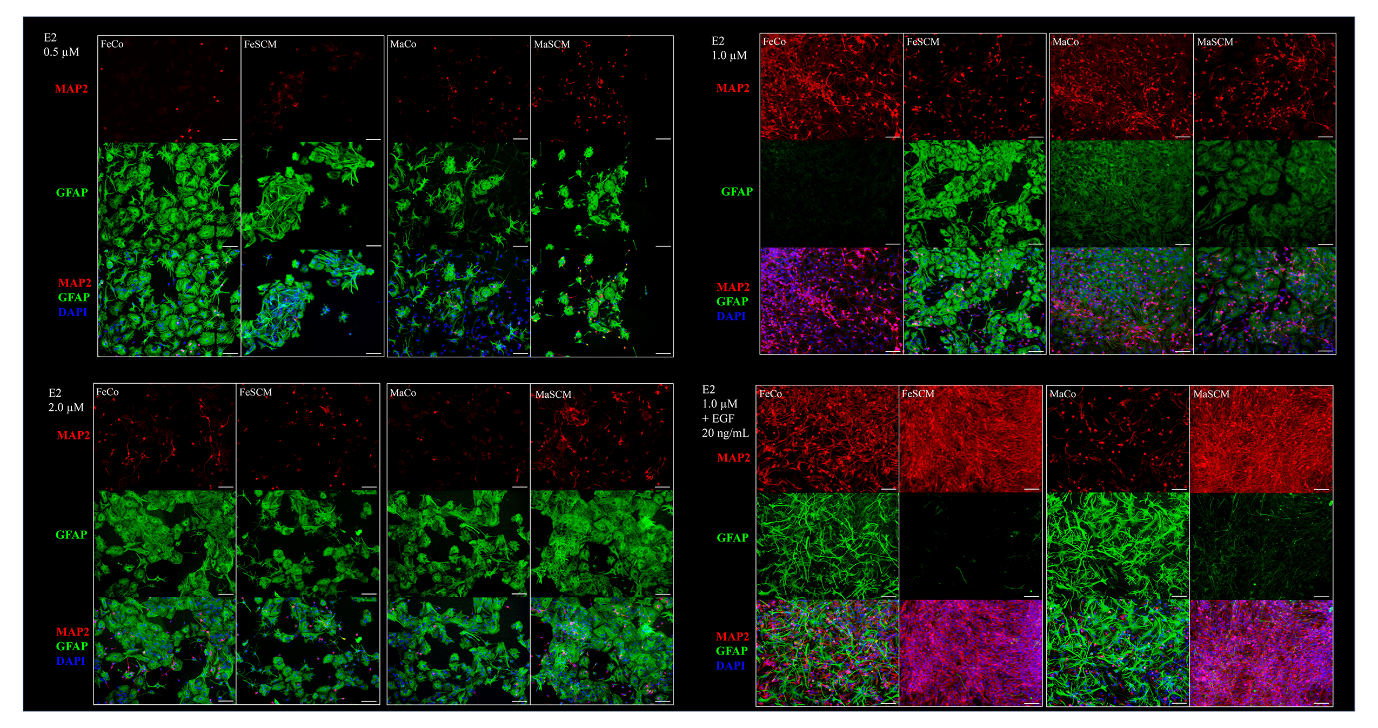


**Figure S5.** Representative fluorescence microscopy images of MAP2-(red) and GFAP-(green) positive cells in SVZ-derived neurospheres cultured with prolactin (PRL) (50 ng/mL, 100 ng/mL, 200 ng/mL and 100 ng/mL with EGF (20 ng/mL) co-treatment). from control (Co) and social cohabitation with mating (SCM) groups in female (Fe) and male (Ma) adult voles. Nuclei were stained with DAPI (blue). Scale bars = 50 µm.


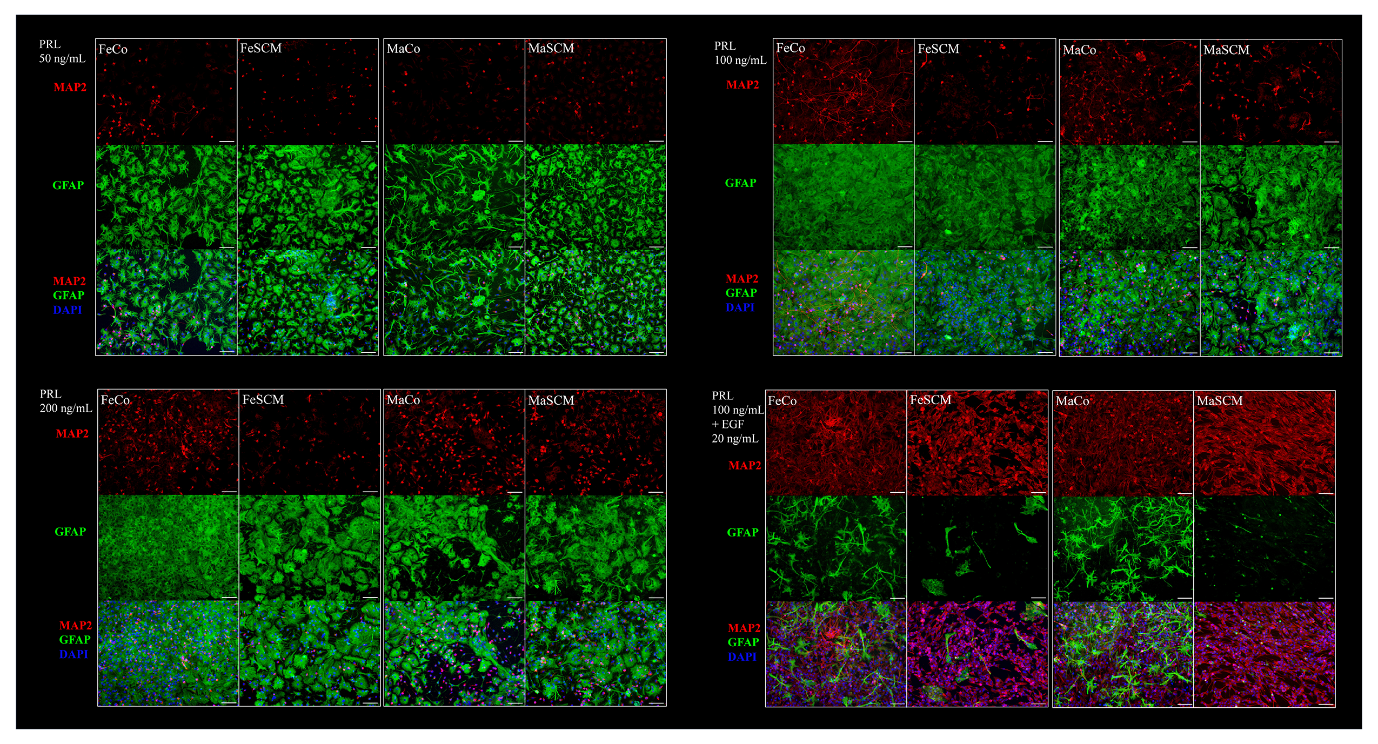


**Figure S6.** Representative fluorescence microscopy images of MAP2-(red) and GFAP-(green) positive cells in SVZ-derived neurospheres cultured with oxytocin (OXY) (0.5 μM, 1 μM and 1 μM with EGF (20 ng/mL) co-treatment) from control (Co) and social cohabitation with mating (SCM) groups in female (Fe) and male (Ma) adult voles. Nuclei were stained with DAPI (blue). Scale bars = 50 µm.


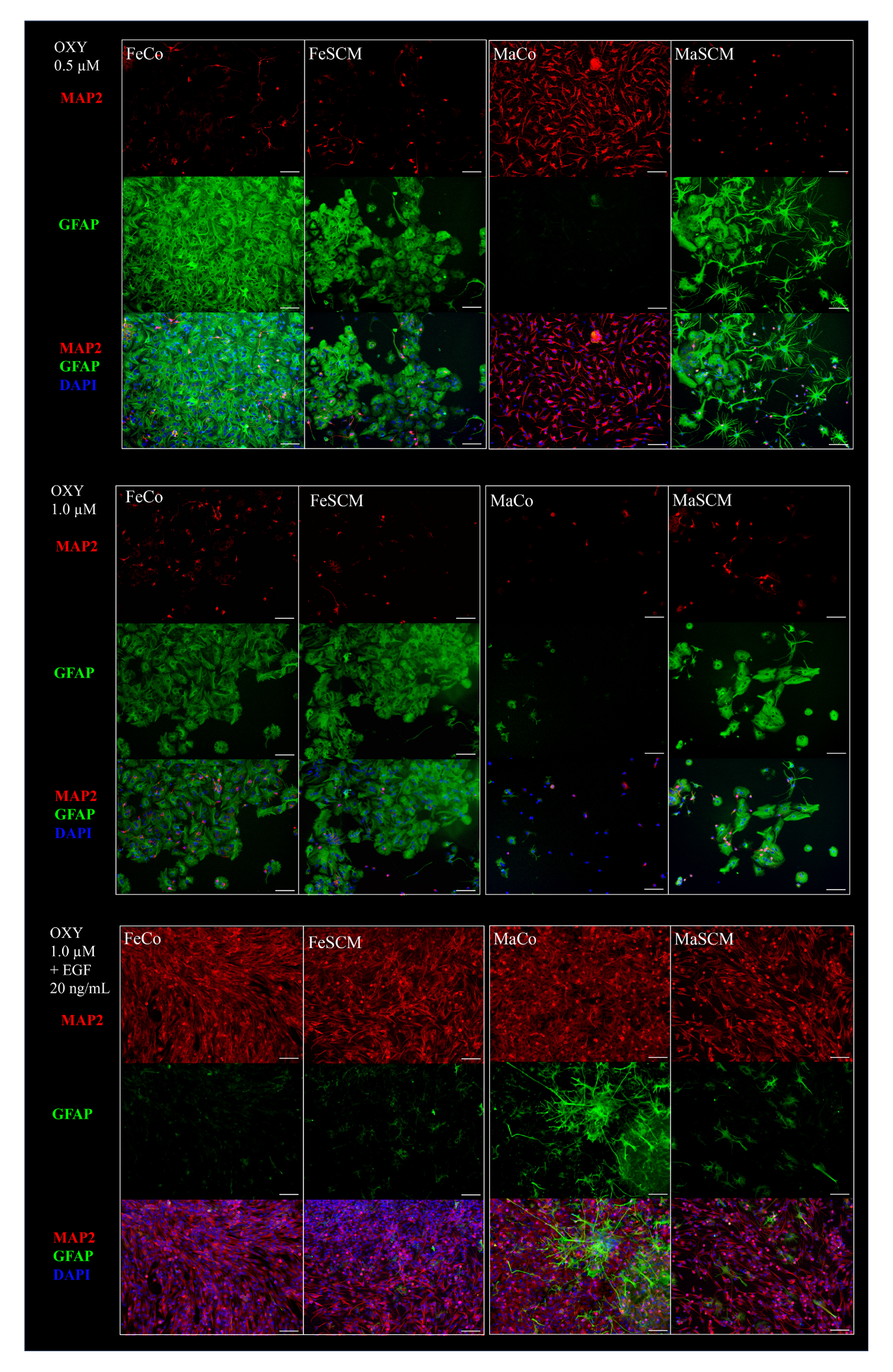


**Figure S7.** Representative fluorescence microscopy images of MAP2-(red) and GFAP-(green) positive cells in SVZ-derived neurospheres cultured with progesterone (P4) (1 μM, 2 μM and 1 μM with EGF (20 ng/mL) co-treatment) from control (Co) and social cohabitation with mating (SCM) groups in female (Fe) and male (Ma) adult voles. Nuclei were stained with DAPI (blue). Scale bars = 50 µm.


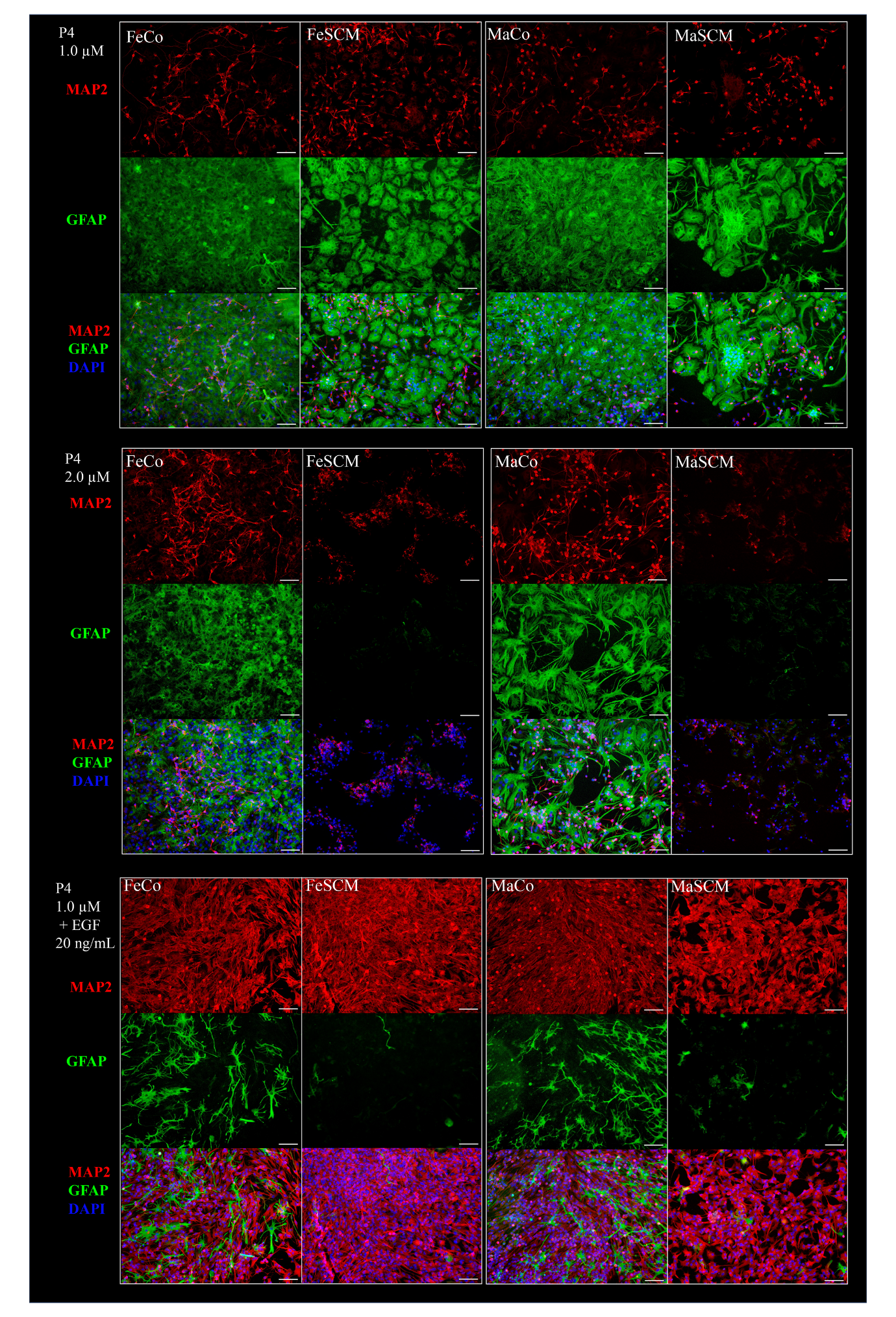

**Table S1.** 2way ANOVA results (sociosexual and sex as factors) and Tukey’s multiple comparisons tests for epidermal growth factor (EGF) treatment. Comparisons between sociosexual groups: FeCo-FeSCM and MaCo-MaSCM (black). Comparisons between sexes groups: FeCo-MaCo and FeSCM-MaSCM (red). Non-significant comparisons were omitted (“----" as without any significance).

**Table S2.** 2way ANOVA results (sociosexual and sex as factors) and Tukey’s multiple comparisons tests for condition without growth factors or any treatments. Comparisons between sociosexual groups: FeCo-FeSCM and MaCo-MaSCM (black). Comparisons between sexes groups: FeCo-MaCo and FeSCM-MaSCM (red). Non-significant comparisons were omitted (“----" as without any significance).

**Table S3.** 2way ANOVA results (sociosexual and sex as factors) and Tukey’s multiple comparisons tests for brain-derived neurotrophic factor (BDNF) treatments. Comparisons between sociosexual groups: FeCo-FeSCM and MaCo-MaSCM (black). Comparisons between sexes groups: FeCo-MaCo and FeSCM-MaSCM (red). Non-significant comparisons were omitted (“----" as without any significance).

**Table S4.** 2way ANOVA results (sociosexual and sex as factors) and Tukey’s multiple comparisons tests for estradiol (E2) treatments. Comparisons between sociosexual groups: FeCo-FeSCM and MaCo-MaSCM (black). Comparisons between sexes groups: FeCo-MaCo and FeSCM-MaSCM (red). Non-significant comparisons were omitted (“----" as without any significance).

**Table S5.** 2way ANOVA results (sociosexual and sex as factors) and Tukey’s multiple comparisons tests for prolactin treatments. Comparisons between sociosexual groups: FeCo-FeSCM and MaCo-MaSCM (black). Comparisons between sexes groups: FeCo-MaCo and FeSCM-MaSCM (red). Non-significant comparisons were omitted (“----" as without any significance).

**Table S6.** 2way ANOVA results (sociosexual and sex as factors) and Tukey’s multiple comparisons tests for oxytocin treatments. Comparisons between sociosexual groups: FeCo-FeSCM and MaCo-MaSCM (black). Comparisons between sexes groups: FeCo-MaCo and FeSCM-MaSCM (red). Non-significant comparisons were omitted (“----" as without any significance).

**Table S7.** 2way ANOVA results (sociosexual and sex as factors) and Tukey’s multiple comparisons tests for progesterone treatments. Comparisons between sociosexual groups: FeCo-FeSCM and MaCo-MaSCM (black). Comparisons between sexes groups: FeCo-MaCo and FeSCM-MaSCM (red). Non-significant comparisons were omitted (“----" as without any significance).
